# Exploring the dog–human relationship by combining fMRI, eye-tracking and behavioural measures

**DOI:** 10.1038/s41598-020-79247-5

**Published:** 2020-12-17

**Authors:** Sabrina Karl, Magdalena Boch, Anna Zamansky, Dirk van der Linden, Isabella C. Wagner, Christoph J. Völter, Claus Lamm, Ludwig Huber

**Affiliations:** 1 Clever Dog Lab, Comparative Cognition, Messerli Research Institute, University of Veterinary Medicine Vienna, Medical University of Vienna, University of Vienna, 1210 Vienna, Austria; 2 Social, Cognitive and Affective Neuroscience Unit, Department of Cognition, Emotion, and Methods in Psychology, Faculty of Psychology, University of Vienna, 1010 Vienna, Austria; 3 Department of Cognitive Biology, Faculty of Life Sciences, University of Vienna, 1090 Vienna, Austria; 4 Information Systems Department, University of Haifa, 3498838 Haifa, Israel; 5 Department of Computer and Information Sciences, Northumbria University, Newcastle-upon-Tyne, NE1 8ST UK

**Keywords:** Neuroscience, Animal behaviour

## Abstract

Behavioural studies revealed that the dog–human relationship resembles the human mother–child bond, but the underlying mechanisms remain unclear. Here, we report the results of a multi-method approach combining fMRI (N = 17), eye-tracking (N = 15), and behavioural preference tests (N = 24) to explore the engagement of an attachment-like system in dogs seeing human faces. We presented morph videos of the caregiver, a familiar person, and a stranger showing either happy or angry facial expressions. Regardless of emotion, viewing the caregiver activated brain regions associated with emotion and attachment processing in humans. In contrast, the stranger elicited activation mainly in brain regions related to visual and motor processing, and the familiar person relatively weak activations overall. While the majority of happy stimuli led to increased activation of the caudate nucleus associated with reward processing, angry stimuli led to activations in limbic regions. Both the eye-tracking and preference test data supported the superior role of the caregiver’s face and were in line with the findings from the fMRI experiment. While preliminary, these findings indicate that cutting across different levels, from brain to behaviour, can provide novel and converging insights into the engagement of the putative attachment system when dogs interact with humans.

## Introduction

The unique relationship between (pet) dogs and their human caregivers bears a remarkable resemblance to the attachment bond of human infants with their mothers: dogs are dependent on human care and their behaviour seems specifically geared to engage their human partner’s caregiving system^1^. Some researchers (e.g.^2–4^) have used concepts and methodologies of the human attachment theory^5,6^ to investigate whether the dog–human relationship conforms to the characteristics of the human attachment bond (reviewed in^7^).

In humans, the original theory of attachment has focused on *parental* attachment, the strong and persistent emotional tie between the child and the caregiver that develops very early in life and serves to protect the child^5,6^. The proximate function is to maintain the proximity between the mother and the child, especially in stressful or dangerous situations^8^. To distinguish true attachment from other affectional bonds, four behavioural criteria were proposed: (a) staying near to and resisting separation from the attachment figure (proximity maintenance), (b) feeling distress upon involuntary separation from the attachment figure (separation distress), (c) using the attachment figure as a base for exploring the environment free of anxiety (secure base), (d) seeking out the attachment figure for contact and assurance in times of emotional distress (safe haven)^9^. A classic test paradigm to characterize attachment relationships is the Strange Situation Procedure (SSP), a set of short episodes of mildly stressful situations of separation and reunion in an unfamiliar environment^10–12^. Comparative psychologists not only have described the similarities of the human mother–child bond and the human–dog relationship^13–15^, but also sought empirical evidence by applying modified versions of the Strange Situation Procedure. Indeed, researchers found clear evidence of all four attachment criteria in dogs^2,3,16–24^. Even more striking, the secure base effect in dogs is specific and tuned to the bond with the caregiver^25,26^.

That the bond between (adult) dogs and the human caregiver is similar to the one between human infants and their mother is an exciting hypothesis, but so far it relies mainly on behavioural and endocrinal evidence. A rigorous test of this hypothesis requires knowledge of the neural networks associated with attachment-related processes. So far, we know that humans share with almost all vertebrates a basic diencephalic and tegmental “social behaviour network”^27^.

Neuroimaging studies of human mothers viewing their children showed that intimate parent–child emotional states are connected to functionally specialized brain areas^28^. This includes, foremost, areas of the so-called limbic system, including the amygdala, the ventral striatum, the ventral tegmental area (VTA), the globus pallidus (GP^29^) and the substantia nigra, as well as the hippocampus^30^. These areas, in humans but also more generally in mammals, are usually associated with affective processes, and may thus support the activation of human attachment-related functions in parenting. In addition, the orbitofrontal cortex (OFC) and the periaqueductal grey (PAG^31^), the dorsal anterior cingulate cortex (dACC), the anterior insula (AI) and the ventrolateral prefrontal cortex (VLPFC^32,33^) show increased activation in mothers upon seeing their own child. Especially seeing their own child’s smiling face caused increased activation of these mesocorticolimbic reward brain regions in their mothers^34^. Unfortunately, it is not clear if the same brain regions are activated when the child faces its mother.

Several recent studies have investigated how dogs perceive humans, and in particular our faces. These revealed that dogs can assess humans’ attentional states^35,36^, and discriminate their caregiver from another familiar person^37^, or from a stranger^38^; the latter was confirmed by converging evidence from two studies using different methods, combining active choice on a touchscreen device^39^ and passive looking preference using an eye-tracking device^40^. Especially interesting is the dog’s ability to discriminate between positive and negative facial expressions of humans and to react appropriately conferring to the valence of the faces^41–46^ (for review see^47^).

Neuroimaging provides an excellent window into the working brain of humans during perception and the associated mental processes, and this non-invasive approach has now also become available to study dogs and their brains. Training dogs to remain still, wakeful, and attentive during scanning was first achieved a decade ago^48,49^, and soon it became the preferred non-invasive research technique to understand the neural correlates of canine cognitive functions^50–54^ (reviewed in^55^). Six previous studies have already investigated the dog’s brain activities while they watched human faces. While a lack of reporting and analysis standards makes it hard to compare the findings in terms of the precise locations of brain areas that are activated, researchers consistently found areas in the canine temporal lobe that responded significantly more to dynamic^56^ or static^57^ images of human faces than to the respective stimuli of everyday objects, especially showing activations in the temporal cortex and caudate nucleus when viewing happy human faces^58^. Another study^59^ identified separate temporal brain areas for processing human and dog faces. Furthermore, a recent study^60^ investigated whether dogs and humans showed a species- or face-sensitivity when being presented with unknown human and dog faces and their occiputs. In contrast to the human participants, they found that the majority of the visually-responsive cortex of the dogs showed a greater conspecific- than face-preference. Two studies, however, found no difference between faces (humans, dogs) and a (scrambled) visual control stimulus^56,61^. Yet, activity related to internal features of human faces (in contrast to a mono-coloured control surface) in temporo-parietal and occipital regions^61^ could be identified.

The dog’s great sensitivity to the human face, especially when showing emotional expressions, seemed to us a promising starting point for the investigation of the dog’s neuronal processing of their human attachment figure. Would dogs’ brain responses be similar to those of humans when watching videos of their beloved pet^30^? To investigate this, and cross-validate our methods, we chose a multi-method approach. Using the same stimuli and, where possible, the same dog subjects, we combined neuroimaging (Experiment 1), eye-tracking (Experiment 2), and behavioural testing (Experiment 3) to explore the canine attachment system on multiple levels. Neuroimaging allowed us to investigate the neural correlates while dogs perceived their human caregiver in comparison to other humans, eye-tracking provided further insights on how dogs perceived the human models focusing on the dogs’ visual exploration, and preference tests explored the dogs’ spontaneous and unrestricted behaviour towards the human faces. In all three experiments, we presented videos transforming from neutral to either happy or angry facial expressions (continuously called “morph videos”) of their human caregiver (caregiver) and an unfamiliar person (stranger). We used dynamic instead of static stimuli to facilitate face recognition by increasing ecological validity and to increase brain activation by supposedly stronger attention (e.g.^62^). Further, varying emotional facial expressions enabled us to investigate the potential interplay of the attachment system and emotions since both emotions and attachment activate similar brain regions (e.g.^63^ for review of emotion processing areas). Finally, to control for familiarity^26^, our study is the first that presented, in addition to the caregiver, the same expressions of another person well-known to the individual dog (familiar person).

In addition to the three experiments we conducted a caregivers’ survey to assess how many hours the primary caregiver and the familiar person actively spent time with the dog per day during the week and on the weekends and the dogs’ age when adopting them. This aimed at getting a glimpse into the dog–human relationship quality and the dogs’ time living together with the caregiver. The dogs of our study spent almost their entire life together with their caregiver and were also involved in many regular activities with them, e.g. daily walks, dog school training and events, or Clever Dog Lab visits for study participation or intense research trainings over years. We thus expected a secure dog–human relationship, and rather subtle attachment-related individual differences across the dogs; this is why we did not test the dogs specifically with the SSP test setup.

Instead, we designed an experiment whose task setup was very similar to the ones of the other two experiments (fMRI, eye-tracking), including a test arena with two computer monitors on the ground simultaneously displaying the same visual stimuli as in these experiments, but where dogs were allowed to immediately react to the stimuli and move freely during the entire test trials.

Since this is the first fMRI study investigating the neural correlates of attachment in dogs (Experiment 1), we aimed to explore whether dogs, similar to humans, recruit the limbic system (e.g. insula, amygdala, dorsal cingulate gyrus^30,32,33^) and brain regions also associated with reward processing (e.g. caudate nucleus^34^) when viewing their human caregiver compared to a stranger or familiar person. During the eye-tracking tests (Experiment 2), we anticipated that the dogs would fixate and revisit the caregiver stimuli comparatively more on the screen than the other presented human faces. Based on previous behavioural studies^26,37,39,40^, we expected the dogs to show a preference for their caregiver over either a stranger or even another familiar person, but this would vary with the facial emotion expressed. We expected the angry facial expression (negative emotion) to evoke more attention and arousal due to being a potential threat or being connected to former unpleasant experiences with angry humans compared to happy faces (positive emotion). The happy faces we predicted to be perceived more positively and with pleasant expectations, e.g. praise, joy, reward^64^. In the behavioural preference tests (Experiment 3), we expected the dogs to spend more time on the “caregiver’s side” of the test arena, and to prefer to look at and to approach the caregiver stimuli compared to the stranger and the familiar person displayed.

## Results

### Experiment 1 (fMRI task)

First, we explored the main effects of face identity (caregiver, familiar person, stranger) and emotion (happy, angry), and their interaction. Regarding the main effect of emotion, we found differential activation of hippocampal areas with increased activation in the left hippocampus for happy morph videos and in the right parahippocampal gyrus for angry morph videos. The main effect of face identity revealed activation changes in areas such as the insula, the bilateral dorsal cingulate cortex, and the postcruciate gyrus. The emotion × face identity interaction effect revealed a difference in activation when viewing the different human models, depending on the emotion displayed (see Table 1 for details).

**Table 1:** Experiment 1 (fMRI), *N* = 17: Task-related activation during visual stimulation.

Contrast, brain region & HRF	Coordinates			*z*-value	Cluster size
	x	y	z		
**Main effect emotion**					
L Hippocampus (T)	− 6	− 11	10	3.09	5
**Main effect: attachment**					
L rostral composite gyrus (T)	− 15	8	10	4.39	9
Encephalon (white matter)	− 10	− 9	7	3.88	9
L postcruciate gyrus (P)	− 12	3	13	3.86	8
R insula (T)	13	− 8	3	3.34	6
L rostral cingulate (T)	− 3	2	13	3.28	5
R rostral cingulate (T)	4	6	10	3.13	5
R rostral composite gyrus (F)	13	0	10	3.07	7
R insula (T)	14	− 12	6	2.98	5
Interaction: emotion × attachment*					
L postcruciate gyrus (P)	− 10	3	13	3.72	5
R postcruciate gyrus (P)	10	− 2	18	3.61	6
R cerebellum	7	− 29	− 8	3.53	8
Encephalon (white matter)	13	− 2	9	3.50	6
Encephalon (white matter)	− 12	− 11	6	3.20	5
Optical nerve	1	2	− 4	3.19	5
Mesencephalon	− 6	− 14	− 4	3.16	5
R parahippocampal gyrus (T)	10	− 20	− 8	3.01	5

Main and interaction effects were tested for significance with a cluster-defining threshold of *p* < 0.005 uncorrected and a minimum cluster size of 5 voxels. The first local maximum within each cluster is reported; coordinates represent the location of peak voxels and refer to the canine breed-averaged template^65^. The template along with another single dog template^66^ served to determine anatomical nomenclature for all tables. See Table 2 for contrasts most relevant for the present study; Supplementary Table S3 contains further contrasts for full transparency.

*O* occipital lobe, *T* temporal lobe, *P* parietal lobe, *O* occipital lobe, *L* left, *R* right.

**Figure 1:**
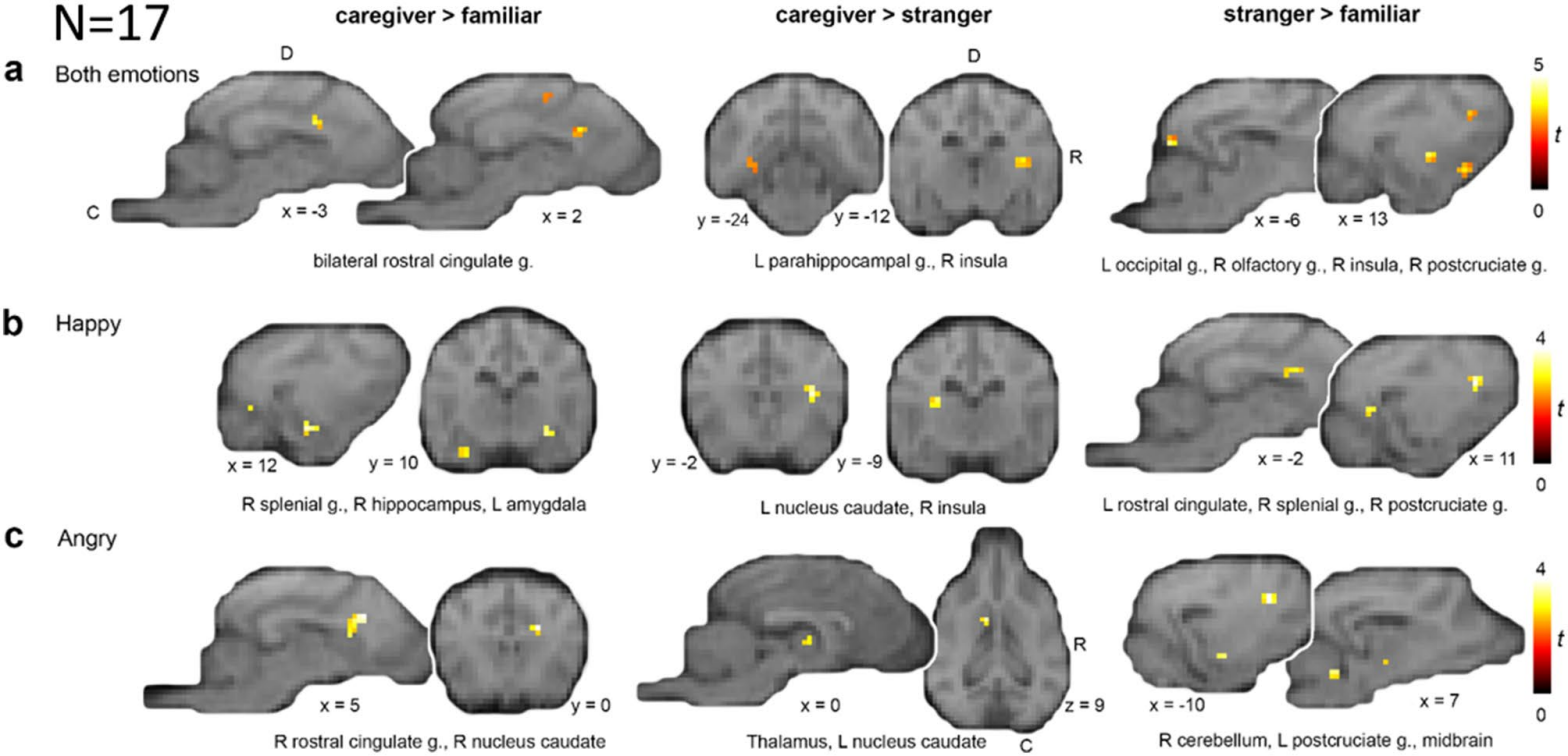
Visual presentation of caregiver (compared to the familiar person or stranger; independent of emotional facial expression) elicited activation increases in areas associated with the attachment system in humans, whereas visual presentation of the stranger (compared to the familiar person) mainly recruited motor and visual processing regions. The caregiver revealed activation in caudate regions for both happy and angry emotional facial expressions. Results are displayed at *p* < 0.005 with a minimum cluster size of 5 voxels (see Table 2 for details), projected onto the mean structural image derived from all dogs. Coordinates refer to the canine breed-averaged atlas^65^. The first sagittal and coronal planes (**a**, first row) and transverse plane (**c**, last row) show the anatomical locations caudal (C), dorsal (D), and right hemisphere (R); all sagittal and coronal planes displayed have the same orientation. Group-based comparison of caregiver against familiar person (caregiver > familiar person), caregiver against stranger (caregiver > stranger) and stranger against familiar person (stranger > familiar person) are displayed (**a**) regardless of emotional facial expression, (**b**) for happy emotional facial expressions, and (**c**) for angry emotional facial expressions. *D* dorsal, *C* caudal, *g*. gyrus, *R* right, *t* t-value.

We further explored the differences in activation depending on the human model regardless of the emotion displayed. In comparison to the familiar person, visual presentation of the caregiver elicited increased activation in brain regions such as the bilateral rostral cingulate, the left parahippocampal gyrus, right olfactory gyrus, as well as rostral temporal and parietal regions (see Supplementary Table S3, for details and Fig. 1). Comparing activation between caregiver and stranger, visual presentation of the caregiver led to increased activation in the bilateral insula, the right rostral cingulate gyrus, the left parahippocampal gyrus, as well as rostral parietal and temporal regions. Viewing the stranger increased activation mainly in the bilateral frontal lobe, brainstem, cerebellum, postcruciate gyrus, and the left insula. When comparing visual presentation of the stranger with the familiar person, we found similar activation patterns but with additional activation in the occipital lobe, the right insula, and temporal regions. Comparing visual presentation of the familiar person to the stranger revealed increased activation in the brainstem and frontal lobe, as well as increased activation in the left cerebellum, right occipital lobe, and the left caudal temporal lobe in comparison to the caregiver (see Supplementary Table S3).

We finally explored the emotion × attachment interaction effect, separately for both happy and angry morph videos, to investigate a potential modulation of attachment due to the two different emotional facial expressions (see Table 2 for details). Regarding the happy morph videos, we observed a similar pattern of activation as described above, but the majority of happy morph videos additionally led to increased activation of the caudate nucleus. Focusing on the visual presentation of human models with angry emotional facial expression, we again observed the same pattern of activation with the angry caregiver eliciting activation in brain regions associated with human attachment processing; but unexpectedly the *angry* caregiver also revealed activation in the caudate nucleus similar to the *happy* caregiver. We focused on the contrasts most relevant for our research question: caregiver > familiar/ stranger, stranger > familiar combined and separate for emotions (see Fig. 1, Table 2); see Supplementary Table S3 for further contrasts in light of full transparency.

**Table 2:** Experiment 1 (fMRI), *N* = 17: Task-related activation during visual stimulation.

Contrast, brain region & HRF	Coordinates			*z*-value	Cluster size
	x	y	z		
**Caregiver > familiar**					
L rostral cingulate gyrus (T)	− 3	2	13	3.80	7
R rostral cingulate gyrus (T)	2	8	12	3.54	12
R rostral suprasylvian gyrus (T)	13	5	12	3.42	8
R lateral olfactory gyrus	14	3	1	3.11	8
L parahippocampal gyrus (T)	− 12	− 24	1	3.00	5
**Caregiver > stranger**					
L insula/white matter (T)	− 10	− 9	7	4.41	18
R rostral composite gyrus (T)	13	0	10	3.65	8
R insula (T)	14	− 12	6	3.57	25
R rostral cingulate gyrus (T)	4	6	10	3.47	9
R medulla oblongata	1	− 50	− 14	3.29	6
L postcruciate gyrus (P)	− 13	6	18	3.24	5
L parahippocampal gyrus (T)	− 13	− 24	1	2.83	6
**Familiar < stranger**					
L occipital gyrus (O)	− 6	− 36	9	4.15	9
R insula (T)	13	− 8	3	3.93	11
Mesencephalon	2	− 14	− 2	3.92	7
L postcruciate gyrus (P)	− 10	3	13	3.90	8
R lateral olfactory gyrus (F)	13	3	− 2	3.43	11
Cerebellum (vermis)	1	− 42	− 5	3.34	10
R medial ectosylvian gyrus (T)	19	− 17	18	3.23	5
R postcruciate gyrus (F)	13	5	15	3.17	5
L caudal suprasylvian gyrus (T)	− 24	− 26	− 1	3.15	6
Optical nerve (T)	1	2	− 4	3.11	9
L rostral ectosylvian gyrus (T)	− 21	− 3	13	2.88	5
**Happy: caregiver > familiar**					
R hippocampus (T)	13	− 14	− 5	3.57	11
L hippocampus (T)	− 6	− 17	12	3.53	11
R presplenial gyrus (P)	2	− 3	22	3.44	8
L caudal cingulate gyrus (T)	− 10	− 21	13	3.01	8
L amygdala/hippocampus (T)	− 13	− 9	− 10	2.97	9
R occipital gyrus (O)	13	− 30	1	2.83	5
L medial ectosylvian gyrus (T)	− 16	− 14	10	2.81	6
**Happy: caregiver > stranger**					
R insula/white matter (T)	13	− 2	9	3.63	10
L rostral ectosylvian gyrus (T)	− 12	− 5	9	3.43	8
L splenial gyrus (O)	− 10	− 26	6	3.28	6
L caudate nucleus/white matter (T)	− 10	− 9	7	3.23	7
L proreus gyrus (T)	− 10	5	6	3.18	5
L caudal ectosylvian gyrus (T)	− 15	− 23	9	3.05	5
R rostral sylvian gyrus (T)	16	− 14	6	3.03	11
L rostral cingulate gyrus/genual gyrus	− 1	15	6	2.88	5
**Happy: familiar < stranger**					
R postcruciate gyrus (P)	13	5	15	3.81	14
L rostral cingulate gyrus (T)	− 1	0	10	3.54	10
L caudal suprasylvian gyrus (T)	− 21	− 29	− 1	3.50	7
R splenial gyrus (O)	11	− 27	4	3.12	6
R caudal suprasylvian gyrus (T)	22	− 26	6	2.99	5
R olfactory tuberculum (T)	4	5	− 5	2.89	7
R medial ectosylvian gyrus (T)	16	− 15	16	2.89	5
R piriform lobe/R amygdala (T)	14	− 8	− 10	2.79	5
**Angry: caregiver > familiar**					
R caudal sylvian gyrus (T)	23	− 12	6	3.92	7
R caudate nucleus	4	9	13	3.92	20
L rostral cingulate gyrus	− 3	2	15	3.91	9
R caudate nucleus/rostral cingulate gyrus (T)	8	0	13	3.74	5
Diencephalon	− 6	− 14	− 2	3.59	7
Medulla oblongata	− 1	− 36	− 14	3.37	11
R medial cingulate gyrus (T)	7	− 6	15	3.37	5
R parahippocampal gyrus (T)	10	− 20	1	3.16	7
R rostral proreus gyrus (T)	13	3	1	3.11	9
L caudal sylvian gyrus (T)	− 21	− 9	− 1	3.04	8
R cerebellum	8	− 38	− 5	2.99	5
Medulla oblongata	7	− 32	− 10	2.90	7
L splenial gyrus (O)	− 10	− 27	4	2.76	6
**Angry: caregiver > stranger**					
L caudate nucleus (T)	− 4	0	9	3.47	5
L thalamus	− 3	− 8	− 1	3.24	11
**Angry: familiar < stranger**					
L postcruciate gyrus (P)	− 10	3	15	3.85	12
L hippocampus/thalamus (T)	− 1	− 11	9	3.78	17
Diencephalon	8	− 12	− 2	3.76	11
Diencephalon	− 6	− 12	− 2	3.70	17
L proreus gyrus (F)	− 6	17	3	3.38	18
L marginal gyrus (P)	− 4	− 3	25	3.33	5
R cerebellum	7	− 27	− 5	3.33	7
L caudal cingulate gyrus (T)	− 1	3	16	3.07	6
R presplenial gyrus (P)	2	− 3	22	2.77	5

Effects were tested for significance with a cluster-defining threshold of *p* < 0.005 uncorrected and a minimum cluster size of 5 voxels. The first local maximum within each cluster is reported; coordinates represent the location of peak voxels and refer to the canine breed-averaged template^65^. The template along with another single dog template^66^ served to determine anatomical nomenclature for all tables. Contrasts of interest for the present study are reported; see Supplementary Table S3 for further contrasts on light of full transparency.

*O* occipital lobe, *T* temporal lobe, *P* parietal lobe, *O* occipital lobe, *L* left, *R* right.

### Experiment 2: Eye-tracking task

**Figure 2:**
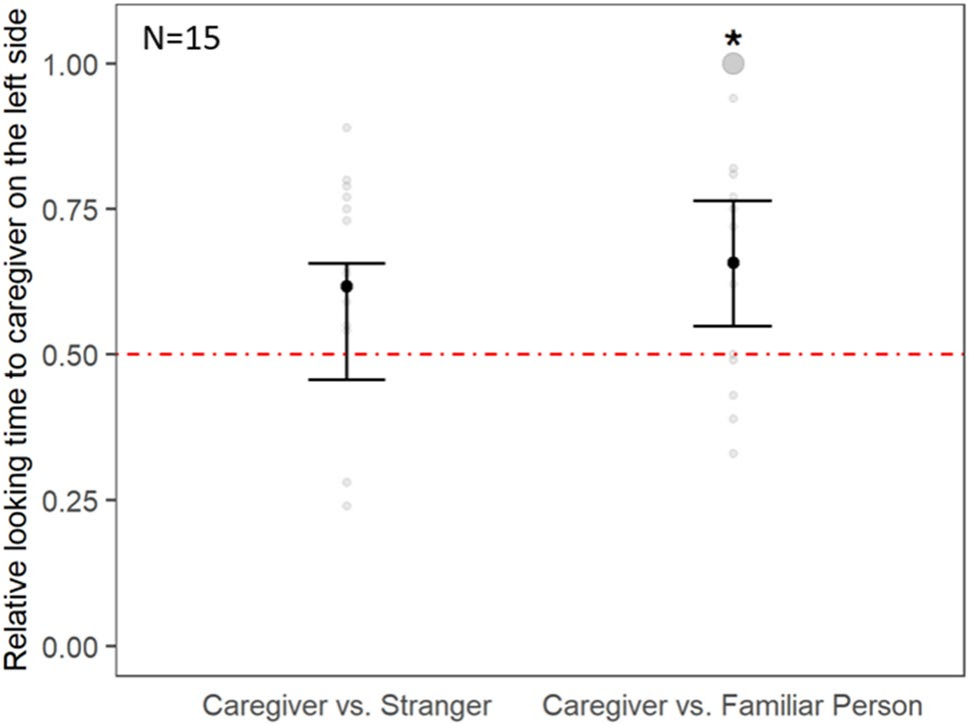
Dogs’ relative looking time to the caregiver presented on the left screen side in Experiment 2a (left; caregiver vs. stranger) and 2b (right; caregiver vs. familiar person). The red dash-dotted line represents the chance level (0.5), GLMM: *p < 0.05. The bootstrapped 95% confidence intervals of the model are indicated by the vertical black lines. The grey points represent the individual looking patterns of each dog per experiment. The size of the points is proportional to the number of individuals.

In Experiment 2a, we presented dogs with morph videos of the caregiver and the stranger side by side. We first analysed the relative looking time to the caregiver. The GLMM model including the predictor variables caregiver location, emotion, trial number, and age did not fit the data significantly better than a null model comprising only the control predictors trial number and age and the random effects (χ^2^ = 2.80, df = 2, *p* = 0.246; Fig. 2). Next, we analysed the latency of the dogs’ first fixation to one of the morph videos. The dogs’ latency for looking at the stranger was significantly shorter than for looking at the caregiver (χ^2^ = 5.75, df = 1, *p* = 0.017; Supplementary Fig. S7, Table S4). The other predictors (emotion, caregiver location, age and trial number) had no significant effect. Finally, we analysed the maximal pupil size. The full model including the predictor variables stimulus, emotion, trial number, and age did not fit the data significantly better than a null model comprising only of the control predictors (age and trial number) and random effects (Supplementary Fig. S9).

In Experiment 2b, we presented dogs with morph videos of the caregiver and the familiar person. For the relative looking time to the caregiver, the full model including the predictor variables caregiver location, emotion, trial number, and age fitted data significantly better than a null model comprising only the control predictors and random effects (χ^2^ = 7.68, df = 2, *p* = 0.021; Supplementary Table S5). We found that when the caregivers were presented on the left side of the screen the dogs looked significantly longer at them than when they were presented on the right side (see Supplementary Fig. S8). The dogs showed a higher relative looking time to the caregiver, i.e. had a significant preference for the left side when the caregiver was displayed on the left side (z = 2.75, *p* = 0.006; Fig. 2). When the caregiver was on the right side, in contrast, dogs did not show a significant preference for either side (z = − 1.71, *p* = 0.087). Emotion, age, and trial number had no significant effect on the dogs’ relative looking time to the caregiver (see Supplementary Table S5). Considering the latency to first fixation, the full model including the predictor variables stimulus, emotion, trial number, and age did not fit the data better significantly than a null model comprising only of the control predictors and random effects. Finally, considering the maximal pupil size, we found that the dogs had a significant larger maximal pupil size when looking at the angry faces of the caregiver and the familiar person compared to their happy faces (Supplementary Fig. S9, Table S6). The other predictors (emotion, age and trial number) had no significant effect on the response of the dogs.

### Experiment 3: Behavioural preference task

We only found tendencies but no significant effects of the stimuli (caregiver, stranger, familiar person) on the dogs’ behavioural responses (two-tailed Mann–Whitney-U-test, *p* > 0.05; see Supplementary Table S9). The descriptive and inferential statistical results are presented in the Supplementary Tables S7–S9.

**Figure 3:**
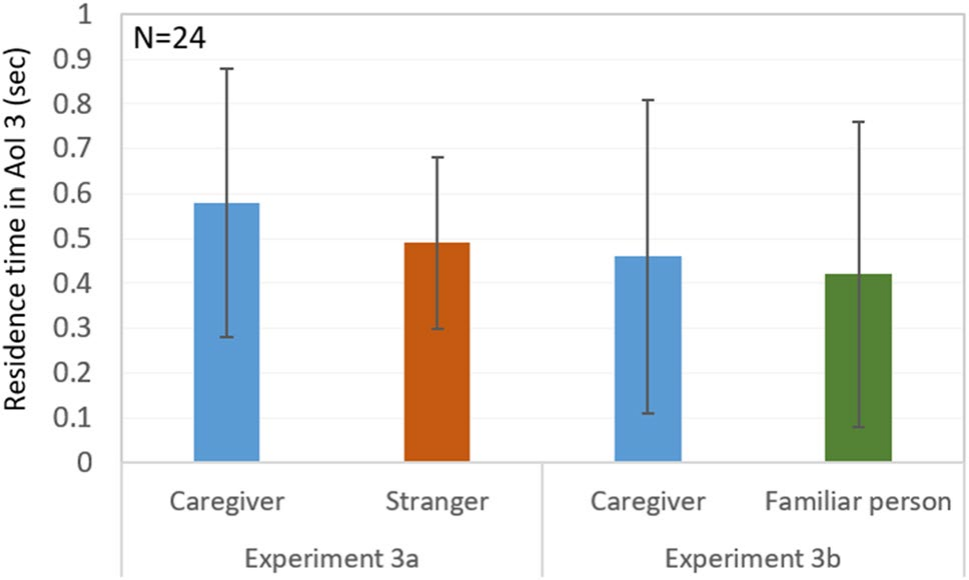
The dogs’ average residence time (in seconds) in the Area of Interest 3 in Experiments 3a and 3b. The 95% confidence intervals are indicated by the vertical black lines. The mean residence time for the caregiver is depicted in blue, for the stranger in dark orange, and for the familiar person in green bars.

In Experiment 3a, we simultaneously presented the faces of the caregiver and the stranger. We found no significant effects, but, on average, the dogs not only tended to spend more time on the caregiver’s side of the test arena than on the stranger’s side, but also spent a longer residence time close to, more time touching (Area of Interest/ AoI 3, see Fig. 3) and more time looking at the screen showing the caregiver than to the screen showing the stranger (for descriptive statistics, see Supplementary Table S7). Of note, when considering the first choices (entering AoI 2, see Fig. S4), we found that slightly more dogs approached the stranger’s face than the caregiver’s face (see Supplementary Table S7). However, in 31 trials (out of *N* = 83) dogs did not enter the AoI 2 at all.

In Experiment 3b, in which we presented the dogs with the caregiver and the familiar person, no significant effects emerged either (Supplementary Table S8). In general, dogs were not very motivated to explore the presented faces. In 51 trials (out of *N* = 83) dogs did not even enter the area at close distance to the screen (AoI 2). Still, the dogs showed, on average, a longer residence time in AoI 1 (arena half) and AoI 3 (close to screen; see Fig. 3) on the caregiver’s side than on the familiar person’s side (AoI 1). Additionally, slightly more dogs went first towards the caregiver’s face than to the familiar person’s face (AoI 2, first choice; see Supplementary Table S8).

## Discussion

In this multi-method approach to investigate the neuro-cognitive basis of the dog–human relationship we analysed the neural, visual and behavioural responses of pet dogs to dynamic images of human faces. We hypothesized that pet dogs would exhibit considerable differences in all three types of responses to seeing the face of a familiar and an unfamiliar human. In addition, on the basis of solid behavioural evidence for a strong, attachment-like bond to their human caregiver, we hypothesized that the dogs would also show a difference between their human caregiver and another familiar, but not attached person, i.e. preferring or more intense (neural, behavioural) responses. Finally, we investigated whether the dogs’ perception of various humans might differ depending on their displayed emotional facial expression. Combining emotions and attachment as experimental factors was intended to explore whether the attachment system for caregivers was activated regardless of their emotion display, or whether positive and negative displays would result in differential activation. Overall, both main hypotheses could be confirmed, although some details of the results, especially regarding the facial expressions, are more difficult to explain.

As expected, the visual presentation of the human caregiver in Experiment 1 led to increased activation in areas associated with emotion and attachment processing (e.g. caregiver > stranger: bilateral insula, rostral dorsal cingulate gyrus, and happy: caregiver > familiar: amygdala^30,63^), and brain regions sensitive to reward processing (e.g. happy: caregiver > happy stranger: caudate nucleus^48,67–70^). This is in line with another dog fMRI study^71^, where the dogs were presented with different scents of themselves, a familiar (not the caregiver or handler) and an unfamiliar human and a familiar and an unfamiliar dog during the scans. The authors found that the olfactory bulb/ peduncle of the dogs was similarly activated by all scents but the caudate nucleus was maximally activated in the familiar human condition. Therefore, it was suggested that the dogs were able to distinguish between the different scents and had a positive association with the one of the familiar human. These findings support our results of caudate nucleus activation when perceiving the human caregiver (for both happy and angry faces; happy: caregiver > stranger, angry: caregiver > familiar, angry: caregiver > stranger) which demonstrates the dogs’ capabilities to identify humans olfactorily and visually and distinguish between them according to their roles in the dogs’ lives. In addition, we observed increased activation in motor (e.g. postcruciate gyrus^72,73^), and further temporal regions, e.g. the rostral suprasylvian and parahippocampal gyrus). Regardless of emotion display, both the hippocampal and rostral cingulate gyrus resulted in increased activation when the dogs saw their primary caregiver in comparison to both the familiar person and stranger (caregiver > stranger, caregiver > familiar). The rostral cingulate gyrus has been hypothesized to play a crucial role for mammalian mother-infant attachment behaviour along with the thalamus (i.e.^77^). In non-human animals, lesions of the rostral and caudal cingulate gyrus result in impairment of maternal behaviour, e.g. in mice^74,75^ rats^76^, and diminished separation cries in squirrel monkeys^77^. In human mothers, watching their child in a stressful situation^31^ or listening to their infant crying^78^ also evoked increased activation in the anterior cingulate cortex among other regions. Further, the rostral cingulate and hippocampal regions along with the bilateral insula and reward regions have also been reported as neural correlates of love^79^, but romantic not maternal love evoked activation in hippocampal regions^80^. The involvement of the parahippocampal gyrus might indicate increased arousal due to memories of the primary caregiver evoked by the presented stimuli^81^ or relatedly increased attention^82^. In the behavioural preference test, we also found slightly more dogs spending a longer residence time on the caregiver’s side of the arena further suggesting a potential increase in attention. But note that increased parahippocampal activation has also been observed in mothers in response to unfamiliar babies compared to their own ones^31^. In contrast, visual presentation of the stranger mainly resulted in increased activation in motor^72,73^ and higher-order visual processing areas (i.e. right medial ectosylvian gyrus^56,59,61,83^). These results might indicate increased motor inhibition^72,84^ and visual attention associated with the salience of a novel and ambiguous (potentially threatening or rewarding) agent. In line with this, we found a shorter latency to look at the stranger in the eye-tracking task (Experiment 2a, caregiver vs. stranger), which supports the possible explanation of a higher attention towards the stranger due to novelty effects^37,85^. Of note, for our fMRI study the dogs were trained to stay motionless in the scanner. However, being exposed to a salient stimulus such as a strange person but also the primary caregiver (caregiver > stranger: precruciate gyrus, premotor cortex) it could have been more demanding for the dogs to stay motionless compared to a familiar person, resulting in an increased motor inhibition (reflected by corresponding differences in motor activation). Lastly, visual presentation of the familiar person elicited no significant difference in comparison to the stranger and, as expected, we did not find any significant activation changes in brain regions associated with attachment processing in humans in comparison to the caregiver for both the stranger and familiar person (caregiver < familiar/ stranger).

Concerning the emotional facial expressions, we found only the caregiver’s face, in contrast to all other presented humans, eliciting similar activation regardless of showing a positive or negative emotion. The display of happy emotional expressions led to activation changes in the caudate nucleus, a brain region previously associated with reward processing (e.g.^86^), and the perception of human faces in dogs^57,58^. Other than that, we observed the same pattern as described above with the happy caregiver eliciting activation in limbic regions (i.e. happy caregiver > familiar: bilateral hippocampus, amygdala; happy caregiver > stranger: R insula, rostral cingulate gyrus) among other regions, i.e. visual cortices (happy caregiver > familiar: R occipital gyrus; happy caregiver > stranger: splenial gyrus). Whereas the happy stranger mainly resulted in increased activation in motor (happy stranger > familiar: R postcruciate gyrus), and visual processing areas (i.e. happy stranger > familiar: R splenial gyrus) but also other (happy stranger > familiar: R amygdala) or similar limbic regions (i.e. R dorsal and rostral cingulate gyrus). However, in comparison to the happy familiar person, visual presentation of the happy stranger additionally resulted in increased activations in limbic regions. Again, the familiar person did not lead to increased activation in regions associated with attachment processing compared to the caregiver (happy: caregiver < familiar).

Regarding negative emotional facial expressions, presenting the angry caregiver surprisingly led to increased activation in brain regions associated with *reward* processing. This might indicate that the attachment figure is positively perceived no matter what emotion he or she shows, and the resulting activation could potentially be related to mechanisms such as (increased) approach motivation^87^. In line with our findings, studies with human mothers, also reported increased caudate nucleus activation in response to a negative emotional display of their own children (e.g.^31^), thus the negative display might elicit an even stronger attachment response. Overall, the angry stranger elicited the strongest activation (highest number of activated clusters) again in mainly motor and visual processing areas, potentially reflecting the further increased salience due to a combination of novelty and a threatening emotional display; however, we did not find increased activation in limbic structures (including the amygdala), as was the case for the angry caregiver. Nevertheless, we did observe increased activation in the L insula and parahippocampal gyrus as well as a visual region (L marginal gyrus). This finding is in line with the unexpected caudate nucleus activation for the angry caregiver, again indicating that solely the primary attachment figure does not elicit a threatening response.

By exploiting the eye-tracking method (Experiment 2) we sought to determine the individual looking patterns of the dogs while they perceived different human faces and facial expressions, especially whether the dogs show specific preferences for the caregivers’ faces. Interestingly, when we confronted the dogs with the simultaneous presentation of the caregiver and the stranger, they showed a quicker first fixation of the stranger’s face. Although this might be seen as contradicting a caregiver’s preference, it could be well explained by novelty effects (e.g.^85,88,89^) or surprise (in human children^90^). As dogs appear to be generally attracted to novel objects in comparison to familiar ones, it is reasonable to assume that seeing the face of an unfamiliar person elicits a first attentional capture, and that this happens irrespective of the displayed emotion. This makes sense from an evolutionary perspective, because it is necessary to rapidly recognize a potential threat, such as a stranger (in chimpanzees^91^, humans^92^). However, this effect seems to be fragile, as it did not survive in terms of longer looking times or pupil size changes. In contrast, a looking time preference was found when we presented the dogs with the faces of their caregiver and a familiar person. Dogs looked longer at their caregiver, but only if her/ his face was presented on the left side of the screen. The fact that the dogs had a general preference for looking on the left side can be explained by a left gaze bias, as was repeatedly found in previous studies^42,46,93^. The left gaze bias we found likely interacted with the caregiver side bias, with the latter being amplified when the caregiver’s face was shown on the left side and being weakened or even extinguished when shown on the right side of the screen. Interestingly, the laterality we found in Experiment 2a was not confirmed in the fMRI or behavioural preference tests but we cannot tell whether this is due to a higher sensitivity to detect such effects in the eye-tracking experiment, or whether it is a false positive. Note that in another recent comparative dog and human fMRI study investigating species- and face sensitivity the authors also did not find any lateralization effects in the dogs in contrast to the human participants^60^.

Concerning the facial expressions, the angry faces, but not the happy ones, of both humans had an increasing effect on the dogs’ pupil size, which is well supported by the literature. Not only does the pupil size provide information about mental activity and attention (see^94^ for review), size changes during stimulus perception reflect emotional arousal related to increased sympathetic activity^95,96^. Only recently enlarged pupil sizes of dogs were found while looking at angry faces compared to happy faces^97^. In addition to emotional arousal, threat- and fear-related stimuli are detected faster (e.g.^98^) and they are also more distracting than positive and neutral stimuli (e.g.^99,100^), likely due to the immediate relevance of such stimuli to survival throughout the evolutionary history.

Although these measurements reflect the dogs’ interest into the different stimuli, we do not know how they interpret them emotionally, i.e. whether the interest is caused by affiliative motivations. We do not even know if the dogs see the stimuli as representations of human faces. Although dogs are capable of recognizing their human caregiver’s face from photographs^101^, and not only discriminate between positive and negative facial expressions of humans but react appropriately to the valence of the faces^41–46,102^, we found no such emotion effect in the dogs when looking at the caregiver and the stranger side by side.

To answer the question how pet dogs behaved when exposed to the stimuli in an unrestrained and more natural setting as fMRI and eye-tracking can offer, we conducted a behavioural preference test (Experiment 3). Several experiments have shown that such a test facilitates the assessment of how dogs react spontaneously to human face stimuli by approaching, avoiding, or ignoring them^37,103–106^. In contrast to Experiment 1 and 2, the dogs could move freely within a test arena that was equipped with two computer screens showing the different faces simultaneously but 135 cm apart from each other (in the left and right half of the arena, respectively). When confronted with the caregiver’s and the stranger’s face at the same time (Experiment 3a), we expected that they would approach the caregiver and avoid the stranger. We found a tendency, although not significant, in support of this expectation. On average, the dogs spent slightly more time on the caregiver’s side of the arena than on the stranger’s side and, more importantly, spent more time closer to the caregiver’s face and more time looking at it, and touched it more frequently and longer than the screen showing the stranger. This trend would be consistent with previous studies comparing the approach/ avoidance behaviour of dogs^24,37–39,107^. However, due to the non-significant results, we cannot derive strong conclusions here. The same is true for the other trend, when considering the first choices, where a few more dogs went towards the stranger’s screen first than to the caregiver’s screen. An explanation could be offered in terms of neophilia and novelty effects^85,88,101^. Dogs might have explored the side with the unknown human first, but after this initial exploration they might have decided to stay closer to their caregiver’s face. In general, the small tendencies, also with regard to the comparison between the caregivers’ and the familiar persons’ faces, may indicate that the dogs, as soon as they made first contact with the computer screens, lost interest. Also the artificial, empty and therefore perhaps scary testing arena might have contributed to these weak effects.

Regarding the limited numbers of trials within the different tasks we were confronted with further study limitations. Initially, we planned to do the same behavioural preference test investigating the effect of the different emotions, i.e. displaying the same human stimuli with different emotions, but during Experiment 3 we found the dogs getting quickly bored in the test arena and partly refused to enter the test arena in Experiment 3b. Therefore, we decided to cancel the testing of the effects of facial emotions. By conducting only few test trials (4 trials per experiment including stimuli repetition to counterbalance the sides), we tried to avoid strong habituation effects. From previous eye-tracking studies^42,108^ in our lab we know that such habituation effects are also relevant for this kind of experiments. During the fMRI scans we tried to balance the amount of necessary stimuli repetitions and the exhausting duration of the scans for the dogs lying motionless in the scanner (two runs of ca. 4.5 min plus an additional minute for preparations (i.e. head localizer scan)). While we consider the number of repetitions per run (30 per emotion, 20 per attachment figure) as sufficient, we would recommend future studies to employ a block design to further increase power and use multiple stimuli per category to prevent potential habituation.

Taken together, the results of the three experiments provide suggestive evidence for attachment-like neural, visual and behavioural responses to the face of the human caregiver. Our results should be treated as preliminary, though, as we decided to choose statistical correction procedures that did not provide strict control of type I errors, but rather aimed to have lower type II error, due to the exploratory nature of our study. For the interpretation of our results, we focused on the clusters in line with findings from previous attachment and emotion processing research and did not discuss all non-expected findings to prevent over-interpretations and speculations. Nevertheless, our aim was to provide other researchers a comprehensive overview of all clusters observed in Experiment 1 to allow future studies probing these results, enabling region of interest analyses based on the coordinates reported in our study and facilitating meta-analyses by providing the unthresholded t-maps on OSF. Thus, future studies should investigate the reproducibility of our results, and potentially expand them to other sensory stimuli (i.e. auditory cues) as well. While the face of a stranger mainly elicited brain activation in motor and higher-order visual processing areas and the face of a familiar person only very weak activations in comparison to both the stranger and the caregiver’s face, the presentation of the human caregiver’s face activated areas associated with emotion and attachment processing. Although the face of a stranger was most attractive at first glance, probably due to novelty effects, a clear preference for the caregiver’s face over the familiar person’s face supported the pattern of brain activations revealed by the fMRI experiment. Finally, the majority of results from the behavioural test showed a larger mean (even though it was not significant) for the dogs’ preference for being close to their caregiver. Still, these findings cannot per se be seen as proof of an attachment relationship, because all our test subjects have lived together with the caregiver for years (see Supplementary Table S1a), which very likely resulted in a positive relationship due to learned associations with rewarding outcomes^106^.

Attachment is defined as an affectional bond with the added experience of security and comfort obtained from the relationship^109^. The attachment system is an innate psychobiological system individuals are born with, not just the result of a collection of rewarding experiences^6^. Like the child-parent attachment the dog-caregiver relationship is not symmetrical, i.e. the attached individual is less cognitively developed and benefits from being attached to a more cognitively sophisticated individual (the mother, the dog’s caregiver), who plays the caregiving role in their relationship^110^.

The activation pattern we found in this study is not specific enough to make a distinction between true attachment and other affectional bonds. In humans, many researchers have investigated the behavioural development of an infant’s ability to recognize faces in relation to infant-mother attachment^111–113^. However, only a limited number of studies have revealed neural correlates of the mother’s face recognition in infants^114^.

In conclusion, this study provides a first attempt to combine three sophisticated methods to improve the understanding of the dog–human relationship. Although each method and experimental setup has its limitations, our converging findings are very promising and set the stage for similar future work. Nevertheless, a great deal remains to be learned about the neurophysiological mechanisms of attachment-like affiliative behaviours in dogs.

## Methods

### Subjects

All subjects were privately owned pet dogs (for details see Supplementary Table S1). The sample of subjects used for the behavioural preference test (Experiment 3) consisted of 24 dogs. Twenty of those had been used for the fMRI task (Experiment 1), and 15 for the eye-tracking test (Experiment 2).

### Dog–human relationship

To evaluate the intensity and probable quality of the dog–human relationship, we conducted a caregivers’ survey (N = 15; 14 females, 1 male) to assess the dogs’ age at the time when they have adopted them and how many hours per day the caregiver and the familiar person (N = 15; 6 females, 9 males) on average actively spent with the dog during the week and on the weekends (see Supplementary Table S1a).

### Ethical statement

**Figure 4:**
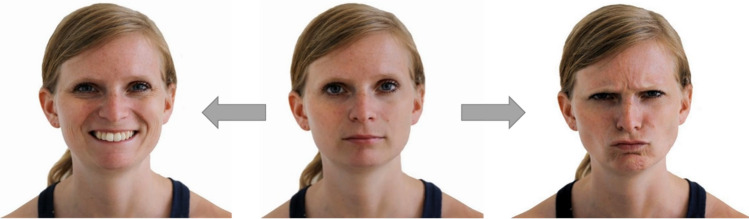
The portrait of a subjects’ caregiver shown with neutral (middle), happy (left) and angry expression (right). The respective video is shown in Supplementary Movie S1, S2.

All reported experimental procedures were reviewed and approved by the institutional ethics and animal welfare committee in accordance with the GSP guidelines and national legislation (ETK-21/06/2018, ETK-31/02/2019, ETK-117/07/2019) based on a pilot study at the University of Vienna (ETK-19/03/2016-2, ETK-06/06/2017). The dogs’ human caregivers gave written consent to participate in the studies before the tests were conducted. Additionally, informed consent was given for the publication of identifying images (see Fig. 4, S2; Supplementary Movie S1, S2) in an online open-access publication.

### Stimuli

We created short (3 s) videos showing human faces that are changing emotional facial expressions (see Fig. 4), transforming (morphing) from neutral to either happy or angry expression (see Movie S1, S2). The face pictures were taken from the human caregiver of each dog, a familiar person, and a stranger (for details, see Supplementary Material).

### Experiment 1: fMRI task

Before the experiment, dogs had received extensive training by a professional dog trainer to habituate to the scanner environment (sounds, moving bed, ear plugs etc.; see^108^). For data acquisition, awake and unrestrained dogs laid down in prone position on the scanner bed with the head inside the coil, but could leave the scanner at any time using a custom-made ramp. The dog trainer stayed inside the scanner room throughout the entire test trial (run) outside of the dog’s visual field. Data acquisition was aborted if the dog moved extensively, or left the coil. After the scan session, the realignment parameters were inspected. If overall movement exceeded 3 mm, the run was repeated in the next test session. To additionally account for head motion, we calculated the scan-to-scan motion for each dog, referring to the frame wise displacement (FD) between the current scan t and its preceding scan t-1. For each scan exceeding the FD threshold of 0.5 mm, we entered an additional motion regressor to the first-level GLM design matrix^115,116^. On average, 3.3% (run 1) and 9.8% (run 2) scans were removed (run 1: ~ 9/270 scans; run 2: ~ 26/270 scans). If more than 50% of the scans exceeded the threshold, the entire run was excluded from further analyses. This was the case for one run (56%/151 scans). We truncated a run for one dog to 190 scans due to excessive motion because the dog was not available for another scan session.

The task alternated between the morph videos (500 × 500 pixels) and a black fixation cross in the centre of the screen that served as visual baseline (3–7 s jitter, mean = 5 s; white background); each run started and ended with 10 s of visual baseline. The presentation order of the morph videos was randomized, but the same human model × emotion combination (i.e., angry stranger) was never directly repeated. The task was split into two runs with a duration of 4.5 min (270 volumes) each, but with a short break in-between if dogs completed both runs within one session. One run contained 60 trials (30 per emotion; 20 trials per human model). Scanning was conducted with a 3 T Siemens Skyra MR-system using a 15-channel human knee-coil. Functional volumes were acquired using an echo planar imaging (EPI) sequence (multiband factor: 2) and obtained from 24 axial slices in descending order, covering the whole brain (interleaved acquisition) using an echo planar imaging (EPI) sequence (multiband factor: 2) with a voxel size of 1.5 × 1.5 × 2 mm^3^ and a 20% slice gap (TR/TE = 1000 ms/38 ms, field of view = 144 × 144 × 58 mm^3^). An MR-compatible screen (32 inch) at the end of the scanner bore was used for stimulus presentation. An eye-tracking camera (EyeLink 1000 Plus, SR Research, Ontario, Canada) was used to monitor movements of the dogs during scanning. The structural image was acquired in a prior scan session with a voxel size of 0.7 mm isotropic (TR/TE = 2100/3.13 ms, field of view = 230 × 230 × 165 mm^3^). Data analysis and statistical tests are described in the Supplementary Material.

### Experiment 2: Eye-tracking task

The eye-tracking task consisted of two tests (Experiment 2a and b) of four trials each, with at least seven days between them. In each trial the morph video of the human caregiver was presented together with either a stranger (Experiment 2a) or a familiar person (Experiment 2b). Both videos were shown with the same, either happy (two trials) or angry (two trials), facial expression. The location (left, right) of the caregiver as well as the emotion (happy, angry) was counterbalanced across the four trials of each test. The dogs went through a three-point calibration procedure first and then received two test trials in a row. At the beginning of each trial the dog was required to look at a blinking white trigger point (diameter: 7.5 cm) in the centre of the screen to start the 15-s (5 × 3 s) stimulus presentation. After a 5–10 min break, this sequence was repeated once. The dogs were rewarded with food rewards at the end of each two-trial block. Data analysis and statistical tests are described in the Supplementary Material.

### Experiment 3: Behavioural preference task

The behavioural preference tests consisted of the measuring of the dogs’ movement patterns inside a rectangular arena facing two videos that were presented simultaneously on two computer screens. The screens were placed opposite to the arena entrance, at a distance of 165 cm on the floor, 135 cm apart from each other (for more details, see Supplementary Material). The dog entered the arena centrally through a tunnel with a trap door and could then move freely for the whole duration of stimulus presentation (10 × 3 s, continuous loop). Like in Experiment 2, the experiment consisted of two tests (Experiment 3a and b) of four trials each, with 1-min breaks between trials and at least seven days between the two experiments. The morph videos were shown in the exact same order and on the same sides (left, right) as in Experiment 2. After each trial, the experimenter called the dog back and went to the corridor outside the test room until the onset of the next trial. The dog was rewarded with a few pieces of dry food at the end of each experiment.

First, we manually cut out the period of stimuli presentation (30 s test trial) from the experiment recordings and then analysed the obtained videos with K9-Blyzer, a software tool which automatically tracked the dog and detected its body parts to analyse the potential behavioural preferences of the dogs towards the different displayed stimuli. Based on the dogs’ body part tracking data (head location, tail and centre of mass in each frame), the system was configured to produce measurements of specified parameters (areas of interest, dogs’ field of view, dog-screen distance) related to the dogs’ stimuli preference. We specified six parameters related to the right and left side/ screen preference (mapped to caregiver, stranger, familiar person), which are described in Supplementary Table S2. The details of the data analysis and statistical values are also provided in the Supplementary Material section.

## Supplementary Information

Supplementary Information

Supplementary vIdeo1

Supplementary vIdeo2

## Data Availability

Supplementary results of Experiments 2 and 3 are included in the Supplementary Material file of this article. Additionally, unthresholded statistical maps from Experiment 1 have been uploaded to OSF.io and are available at osf.io/kagy3.
