# Supplementary material for: Exploring the dog–human relationship by combining fMRI, eye-tracking and behavioural measures
Source: Sci Rep. 2020 Dec 17;10:22273. doi: 10.1038/s41598-020-79247-5 (PMC7747637; doi:10.1038/s41598-020-79247-5)
Supplement: Supplementary file 1 — Supplementary Information [file 41598_2020_79247_MOESM1_ESM.docx]

**Exploring the dog-human relationship by combining fMRI, eye-tracking and behavioural measures**

Sabrina Karl, Magdalena Boch, Anna Zamansky, Dirk van der Linden, Isabella C. Wagner, Christoph J. Völter, Claus Lamm, and Ludwig Huber

**SUPPLEMENTARY MATERIAL**

**METHODS**

**Subjects**

All subjects were privately owned pet dogs recruited from human caregivers in Vienna and nearby via the Clever Dog Lab website and database. They were of different breeds, both sexes, and their age ranged from two to eleven years (see Table S1). The sample of subjects used for the behavioural preference test (Experiment 3) consisted of 24 pet dogs (Table S1). Only a subset of this sample had been used for the fMRI task (Experiment 1). It consisted of 20 dogs (Table S1). One dog (Joy) only performed one of two experiment trials due to a cruciate ligament rupture during the period of data collection and three dogs (Frozen; Lenny, Tini) were excluded from the data analysis because of epilepsy or enlarged ventricles. Hence, the data of 17 dogs was analysed in the end. The sample of the eye-tracker test (Experiment 2) consisted of 15 dogs (Table S1).

| no. | dog | breed | sex | age in 2020 (in years) | fMRI morph task - tested in (year) | Behavioural preference test - tested in (year) | Eye-tracker test - tested in (year) |
| --- | --- | --- | --- | --- | --- | --- | --- |
| 1 | Aeden | Border Collie | m | 12 | 2019 | 2018 | 2019 |
| 2 | Amy8 | Border Collie | f | 10 | 2018 | 2018 | 2019 |
| 3 | Apryl | Border Collie | f | 11 | X | 2018 | X |
| 4 | Balian | Border Collie | m | 10 | X | 2018 | 2019 |
| 5 | Black Velvet | Labrador | f | 5 | 2018 | 2018 | X |
| 6 | Cameron | Border Collie | m | 8 | 2019 | 2018 | 2019 |
| 7 | Carlisle | Border Collie | m | 9 | 2019 | 2018 | 2019 |
| 8 | Chasie | Border Collie | f | 11 | 2019 | 2018 | 2019 |
| 9 | Cliff | Border Collie | m | 6 | 2019 | 2018 | X |
| 10 | Emily | Border Collie | f | 12 | 2018 | 2018 | 2019 |
| 11 | Frozen | Labrador | f | 12 | 2018* | 2018 | X |
| 12 | Gatsby | Border Collie | m | 10 | 2019 | 2018 | 2019 |
| 13 | Joy | Labrador | f | 11 | 2019 | 2018 | X |
| 14 | Kayleigh | Border Collie | f | 9 | 2019 | 2019 | 2019 |
| 15 | Kiki | mixbreed | f | 5 | 2019 | 2018 | X |
| 16 | Lenny | Border Collie | m | 11 | 2018* | 2018 | 2019 |
| 17 | Linus | Australian Shepherd | m | 6 | 2019 | 2018 | 2019 |
| 18 | Luise (deaf) | mixbreed | f | 6 | 2019 | 2019 | X |
| 19 | Maeva | mixbreed | f | 9 | 2018 | 2018 | 2019 |
| 20 | Miley | Border Collie | f | 11 | 2018 | 2018 | 2019 |
| 21 | Sunny | Border Collie | f | 3 | X | 2018 | X |
| 22 | Tiara | Border Collie | f | 7 | 2019 | 2019 | 2019 |
| 23 | Tini | Boxer | f | 9 | X | 2018 | X |
| 24 | Ziva | Border Collie | f | 9 | X | 2019 | 2019 |

**Table S1.** Characteristics and experimental assignment of subjects.

Note. *excluded from fMRI data analysis; X stands for not participated in this test.

**Table S1a.** Data from a caregivers’ survey about the dogs’ age when being adopted, the average active interaction times of the dogs with their primary caregivers and the familiar persons and the time living together with the caregiver.

**Stimuli**

Each dog was presented with the portraits of her/ his human caregiver, of another familiar person and of an unknown person. As familiar person we chose a person the dogs were highly familiar with and had interacted with repeatedly in the past. In most cases this was the human partner of the primary caregiver (attachment figure) who lives in the same household. In some cases, the displayed familiar person was the mother, daughter, niece or a close friend of the primary caregiver (see Table S1a). Our rationale for this was based on a previous study of our lab in which we found the attention of dogs towards humans depending on their relationship, not only on social familiarity^1^. Therefore, we expected the dogs to be less attached to the familiar person in comparison to the primary caregiver and presented them to rule out familiarity of the displayed stimuli. Four human models served as strangers (50% female, age-matched to caregivers/ familiar persons) and were randomly assigned to each dog. Instead of static images we created short (3 s) videos morphing into two facial expressions of the same face. Morph videos were created using the commercial software Abrasoft FantaMorph (<https://www.fantamorph.com/>).

Of each face we used four images where the human expressed neutral, angry, and happy (with and without teeth) facial expressions. Both angry and happy morph videos started with the neutral facial expression morphing into one of the emotional facial expressions (2 s morph, 1 s static display). The set of morph videos (caregiver, stranger, familiar person) each dog saw was luminance-controlled using the Matlab-based SHINE toolbox^2^ and they morphed with the same parameters to control for motion. Additionally, we took all portraits in comparable light conditions. Furthermore, we ran an additional analysis across all portraits; angry and happy morphs do not differ in hue (t(59) = -1.14, p = 0.26), saturation (t(59) = 1.21, p = 0.23) and contrast (t(59) = 0.04, p = 0.97).

To ensure that emotions were actually recognized as happy or angry, and that they were perceived as realistic, we conducted a short online survey with human participants (*N* = 26, 14 females, mean age = 31.92, *SD* = 12.73) rating happiness, anger, realism, and arousal on a visual-analogue scale ranging from 0 (not at all) to 100 (extremely). The anger ratings were significantly higher for angry (mean = 62.10, *SD* = 24.08) compared to happy facial expressions morph videos (mean = 9.04 *SD* = 11.67; *t*(25) = 58.60, *p*< 0.001), and the happiness ratings were significantly higher for the happy morph videos (angry morph videos: mean = 14.16, *SD* = 15.11, happy morph videos: mean = 79.31, *SD* = 16.41; *t*(25) = 85.59, *p*< 0.001). Both conditions were rated as being perceived realistically (realism ratings were both above 50; angry morph videos: mean = 53.51, *SD* = 28.98; happy morph videos: mean = 72.61, *SD* = 25.62), although ratings appeared significantly higher for the happy compared to the angry morph videos (*t*(25) = 15.41, *p*< 0.001). Arousal was generally low (angry morph videos: mean = 25.12, *SD* = 23.12; happy morph videos: mean = 19.12, *SD* = 19.97), but was rated as significantly higher for the angry compared to the happy morph videos (*t*(25) = 7.60, *p*< 0.001).

**Experiment 1: fMRI task**

**Data analysis**

Imaging data was analysed using SPM12 (https://www.fil.ion.ucl.ac.uk/spm/software/spm12/) and Matlab 2014b (MathWorks). First, we performed slice timing correction^3^ with the middle slice as reference and image realignment, then we manually reoriented the functional images and structural image to match the orientation and origin (rostral commissure) of the canine breed-averaged template^4^ using the SPM “Display” tool. Next, we skull-stripped the structural image using individual binary brain masks created with itk-SNAP^5^ with the SPM “ImCalc” function. Then, we co-registered the structural image, the binary brain mask, and functional imaging data to the mean image of both runs. After segmentation of the structural image into grey matter, white matter, and cerebrospinal fluid, we normalized the functional and structural imaging data, along with the binary brain mask. Next, we resliced (1.5 mm isotropic) and smoothed the functional imaging data using a 3 mm Gaussian kernel (full-width-at-half-maximum, FWHM). Finally, we normalized the labels from another canine template^6^ to the breed-average template space^4^, enabling a more detailed description of brain areas (see also^7^ for detailed description of the pre-processing workflow). The translation threshold (translational displacement along x, y, z-axes) served as rough cut-off to decide if a run has to be repeated in a subsequent session. We did not specify a threshold for rotational displacement (pitch, raw, and roll). However, to additionally account for head motion, rotational displacements were converted from degrees to millimetres and we then calculated the scan-to-scan motion for each dog, referring to the frame wise displacement (FD) between the current scan t and its preceding scan t-1. Thus, frame wise displacement accounts for both rotational and translational displacements.

Data analysis was performed using a general linear model (GLM) approach. The first-level matrix of each dog contained 6 task regressors: happy/angry caregiver, happy/angry familiar person, happy/angry stranger. All trials were time-locked to the onset of each event, estimated as a boxcar function (duration: 3 s) and convolved with a haemodynamic response function (HRF) specifically tailored for canine fMRI^7^. As additional nuisance regressors, we included the six realignment parameters along with regressors modelling frame wise displacement (see above). We used normalized and individual binary brain masks (see above), and a high-pass filter with a cut-off at 128 s was applied.

For group analysis, all contrasts were entered in a 2 x 3 flexible factorial design with emotion (happy, angry) and attachment (caregiver, familiar person, stranger) as within-subject factors. Conditions were compared using post-hoc paired-sample *t*-tests. Activation was tested for significance using cluster-inference with a cluster-defining threshold of *p*< 0.005 and a minimum cluster size of 5 voxels. Given the exploratory character of our study, we chose a threshold trying to find a good balance between false positive and false negative results. Lowering the threshold would have resulted in a substantial increase of clusters, predominantly with a cluster size of 1-2 voxels. Increasing the threshold, the number of clusters would have substantially decreased, but considering the small size of subcortical (limbic) regions, we did not want to risk excluding meaningful activation. Thus, we chose the minimum cluster threshold of 5 voxels for pragmatic reasons to both allow an exploration of the activation pattern on a whole-brain level but restrict the number of false negatives.

**Experiment 2: Eye-tracking task**

**Apparatus**

The eye-tracker test took place in the eye-tracking room of the Clever Dog Lab (for detailed setup and equipment see^8^). We used the eye-tracking system EyeLink 1000 (SR Research, Ontario, Canada) to record the monocular eye movements of the dogs’ right eyes. For more details about the eye-tracker see^9^. The setting included a chin rest to stabilize the dog’s head and a computer screen (27 inch, ASUS PB 278) to present the visual stimuli simultaneously side by side (1024 x 768 pixels). The screen was placed 50 cm in front of the dogs’ eyes and the centre of the screen was on the same level as the dogs’ eye height. The scene area of the screen was 45.3 cm x 34 cm (4:3 format) and the stimuli were displayed full-sized centrally on each half of the scene area. During the test trials the dogs could either sit or stand (see Fig. S1). The chin rest, the height and the angle of the eye-tracker were adjusted for each subject.


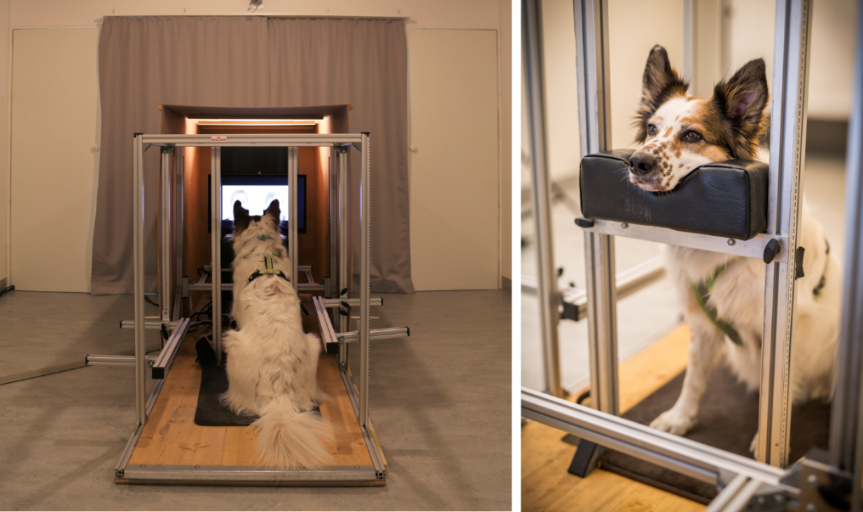


**Figure S1.** Dog subject in the eye-tracking apparatus from behind (left) and from the front (right).

**Procedure**

The eye-tracker task consisted of two experiments (Experiment 2a and b) of four trials each, with at least seven days between them. In each trial the morph videos of the human caregiver together with either a stranger (Experiment 2a) or a familiar person (Experiment 2b) with either happy (two trials) or angry (two trials) facial expression were shown. The location (left, right) of the caregiver as well as the emotion (happy, angry) was counterbalanced across the four trials of an experiment.

During the experiments the dogs went through a three-point calibration procedure first and then received two test trials in a row. At the beginning of each trial the dog was required to look at a blinking white trigger point (diameter: 7.5 cm) in the centre of the screen to start the 15- (5 x 3 s) stimulus presentation (trigger point disappeared when trials began). After a 5-10 min break, this sequence was repeated once. The dogs were rewarded with food rewards (2-3 pieces of sausage) at the end of each two-trial block.

**Data analysis**

To analyse the eye-tracking data we created two areas of interest (AoI), including the presented humans’ faces, necks and parts of the shoulders (see Fig. S2), using EyeLink Data Viewer (version 3.2.48; SR Research Ltd.). The size of each determined AoI was 25.5 cm x 17.7 cm.


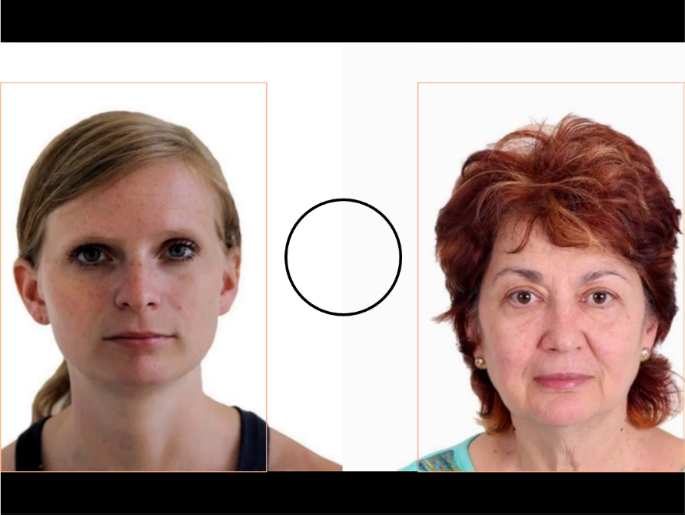


**Figure S2.** Scheme showing the AoIs for the data analysis (orange boxes) and the trigger point area (black circle) in the middle of the screen of the eye-tracker test.

To investigate the dogs’ viewing patterns and whether they showed a preference towards the different visual stimuli we analysed the relative looking time to the caregiver (dwell time into the caregiver AOI divided by the sum of the dwell times into both AOIs). We used the relative looking time as a measure of dogs’ preference for their caregiver (1 = dogs only look at the caregiver; 0 = dogs only look at the other human). To analyse the effects of the caregiver location and emotion on the dogs’ relative looking time, we used beta distributed Generalized Linear Mixed Models (GLMM^10^). We also included trial number and age as control predictors. We included all of these factors as fixed effects, subject ID as a random effect and all possible random slope components except for the correlation parameters between random intercepts and random slopes (GLMM 1: caregiver vs. stranger, GLMM 4: caregiver vs. familiar person^11,12^). To test for deviations from the hypothetical chance level of 0.5, we centred all predictor variables except for the test variable. In this case, the intercept reveals whether the reference category of the test predictor deviated from chance (0.5). Overdispersion (overdispersion parameter - GLMM 1: 0.88, GLMM 4: 1.02) and collinearity (variance inflation factor (VIF) - GLMM 1: 1.00-1.01, GLMM 4: 1.00-1.03) appeared to be no issues.

To examine the latency of the first fixation into one of the two AoI (human face stimuli) per trial and the maximal pupil size of the dogs during the stimulus presentation we used gamma distributed GLMMs (^13^; GLMM 2, 3: caregiver vs. stranger; GLMM 5, 6: caregiver vs. familiar person). We used the same predictor and control variables as in GLMM 1 and 4. We included the subject ID and trial ID as random effects as well as all possible random slope components except for the correlation parameters between random intercepts and random slopes. Overdispersion (GLMM 2: 0.46, GLMM 3: 0.07, GLMM 5: 0.65, GLMM 6: 0.13) and collinearity (VIF: GLMM 2: 1.01-1.08, GLMM 3: 1.00-1.01, GLMM 5: 1.00-1.05, GLMM 6: 1.01-1.02) were no issues.

As an overall test of the effect of the predictor variables, we initially compared the fit of the full model with that of a null model (i.e. a model including only the control variables and the same random effects structure^11^; R function drop1 with argument 'test' set to "Chisq") based on a likelihood ratio test (LRT^14,15^). All models were fitted in R (version 3.6.1^16^) using the function glmmTMB provided by the R package glmmTMB (version 0.2.3^17^) except for GLMM 3 due to convergence issues. In this case, we used the function glmer provided by the R package lme4 (version 1.1.21^18^). We obtained the confidence intervals (CI) by using 1,000 parametric bootstraps and also bootstrapping over the random effects. The model stability we assessed by dropping the levels of the random effects one at a time and comparing the estimates derived with those obtained for the full data set which revealed no influential levels of the random effect. The model appeared to be stable in terms of the fixed effects.

**Experiment 3: Behavioural preference task**

**Stimuli**

The morph videos were presented on two computer screens (27 inch, ASUS PB278Q) that were placed opposite to the arena entrance (distance: 165 cm) on the floor, 135 cm apart from each other (see Fig. S3). The scene area of each screen and accordingly the size of the presented stimuli was 45.3 cm x 34 cm (4:3 format).


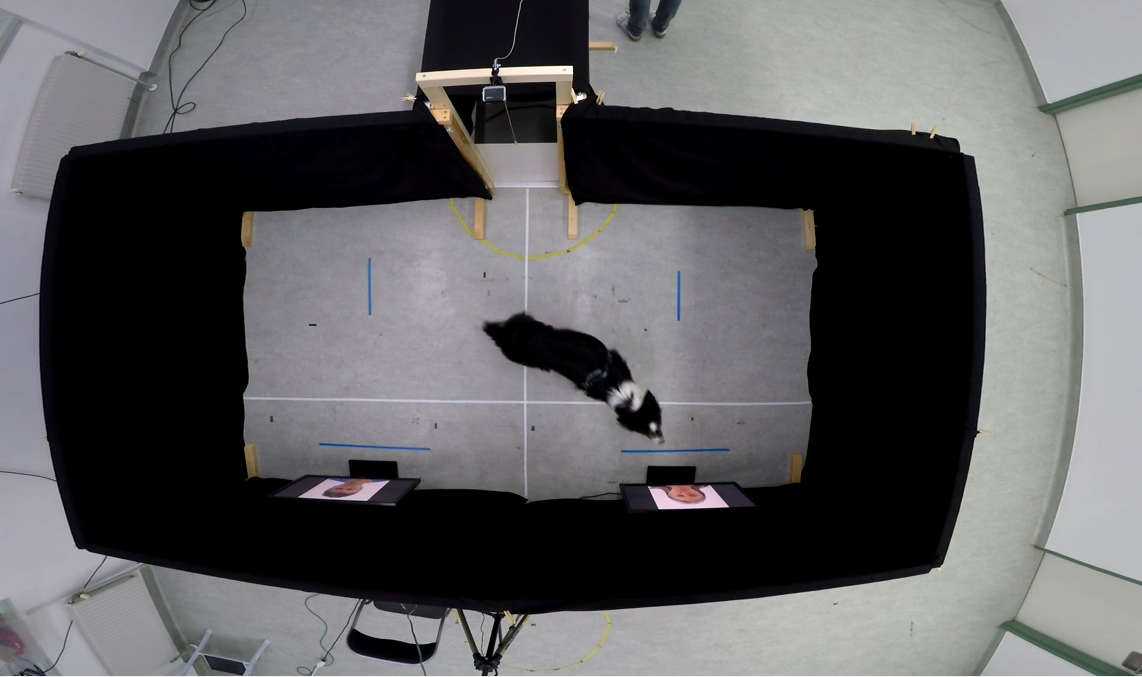


**Figure S3.** View from the ceiling above the arena (Camera 1). Dog freely moving in the arena for the behavioural preference test. Experimenter 1 stands to the left of the tunnel.

**Apparatus**

The behavioural preference experiment was carried out in a rectangular arena (335 cm x 180 cm x 108 cm) built of wooden fences (see Fig. S3) inside a large test room (700 x 600 cm) of the Clever Dog Lab. All fences were covered with black fleece blankets to prevent the view to the outside. The dogs entered the arena through a tunnel (120 cm x 37 cm x 108 cm; covered fences as well) which could be closed by a trap door from behind. An action camera (CAM1; GoPro Hero7 Black) installed in the middle of the ceiling above the arena was used to record the dogs’ behaviour during the tests. A second action camera of the same type (CAM2) was centrally attached above the trap door. It was used by a second experimenter outside the test room to detect when the dog’s head appeared upon entering the arena and to start the stimulus presentation.

**Data analysis**

Overall, 192 test trials (recordings), i.e. respectively 96 test trials in Experiment 3a (caregiver vs. stranger) and b (caregiver vs. familiar person), were analysed. In both Experiments, 26 trials (Experiment 3a: *N* = 13; 3b: *N* = 13) were invalid due to no stimuli perception by the dogs, e.g. because of directly turning away after entering the test arena, and had to be excluded from the data analysis. We used the K9-Blyzer software tool^19,20^ for automatic analysis of the experiments’ video footage, adjusting specific features for the measured parameters, e.g. dogs’ side or stimuli preferences, during the test trials. The average percentage of the dog centre of mass detection per video was 99.5% and the percentage of the tip of the nose detection was 95% (measurements based on the neural net models rate metric). Prior to the automatic data analysis, K9-Blyzer was validated by comparing the system analysis of two parameters (dogs’ first choices; screen touches with the nose) to the manual test video coding conducted with the software Solomon Coder (version beta 19.08.02; developed by András Péter, [www.solomoncoder.com](http://www.solomoncoder.com)), reaching accuracy of 96%.

First, we investigated the effect of the stimuli presentation on the left or right side of the test arena on the dogs’ behavioural responses for both experiments (3a: caregiver vs. stranger, 3b: caregiver vs. familiar person; two-tailed Mann-Whitney-U-test). Following, we examined a potential caregiver preference of the dogs regardless of the side the caregiver was presented on (two-tailed Mann-Whitney-U-test).

To investigate the dogs’ preference behaviours, three AoIs of varying size within the test arena were determined, i.e. the test arena side, including one screen (AoI 1), one area to detect the dogs’ first stimulus choice and screen proximity (AoI 2) and the dogs’ direct approach of a screen by touching it with the nose (AoI 3; see Table S2). Therefore, we measured the dogs’ time spending in each AoI. We analysed the dogs’ first choice by checking which of the AoI 2 (left or right side) was entered first within a test trial. Furthermore, we gauged the average distances between each dog and the two separate screens throughout each test trial. To examine the dogs’ attention and possible interest in the stimuli, i.e. the screens, per test trial, we used the Field of View (FoV) feature of the K9-Blyzer software to estimate the dogs’ FoV, i.e. stimuli perception field, and to detect their looking time at one screen within this approximated FoV (see Table S2).

| **Areas and measure of interest** | **Measured parameters per test trial** | **Description of dogs’ behaviour and measured parameter** |
| --- | --- | --- |
| AoI 1: Left or right test arena side | Dogs’ residence time in AoI 1 (in s) | Time span each dog spent in the left and/ or right AoI 1. We measured the dog’s centre of mass, i.e. centre of the tracking box around the dog, being within AoI 1 (see Fig. S4). |
| AoI 2: Big area (165 cm wide) around the left or right screen (frontal screen distance: 45 cm) | First choice (counts) | The dogs’ first entry into the left or right AoI 2. We measured the dog’s head centre of mass within AoI 2 (see Fig. S4). |
| AoI 2 - screen proximity | Dogs’ residence time in AoI 2 (in s) | Time span each dog spent in the left and/ or right AoI 2. We measured the dog’s centre of mass within AoI 2 (see Fig. S4). |
| AoI 3: Small area (screen width/ 65 cm wide) around the left or right screen (frontal screen distance: 15 cm) | Dogs’ nose detection time in AoI 3 (in s) | Time span the dog’s nose was detected in the left and/ or right AoI 3 (see Fig. S4, S5). We measured the tip of the nose (using a neural net model trained in the DeepCutLab Framework^21^) within AoI 3. |
| FoV | Dogs’ time looking at one screen (in s) | Time span when one screen was fully detected in the approximated FoV (stimuli perception field) of the dog (see Fig. S5). |
| Distance between the dog and each screen | Dogs’ average distance to each screen (in pixels) | Average distance between the dog and each screen (see Fig. S6). |

**Table S2.** Description of the three AoIs in the test arena, the measured parameters and the dogs’ shown behaviours during the test trials.

**
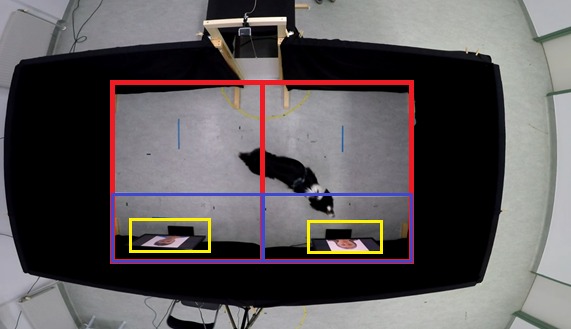
**

**Figure S4.** View from the ceiling (CAM1). Dog moves freely. The coloured frames show the three analysed Areas of Interest: AoI 1 (red), AoI 2 (blue) and AoI 3 (yellow).

**
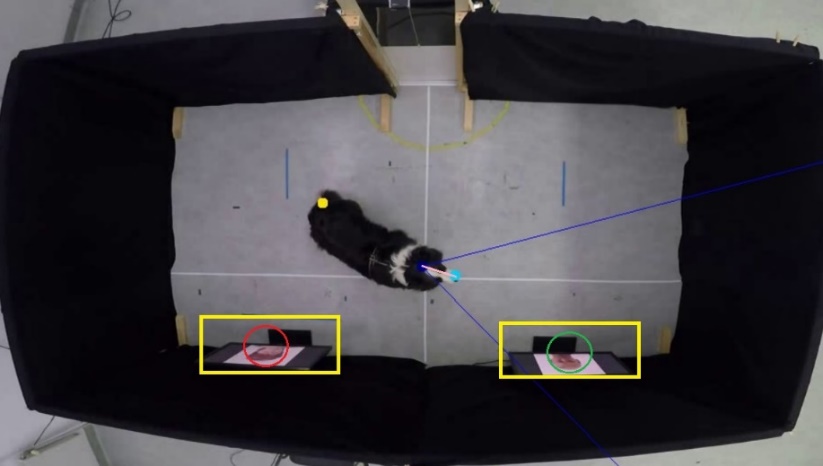
**

**Figure S5.** The two blue lines from the dog’s centre of the head constitute the approximated FoV (60^o^ - 30^o^ on each side of the orange face line). The left screen (from dog’s perspective; marked by a green circle) is detected within the determined FoV, while the right one (red circle) is not.


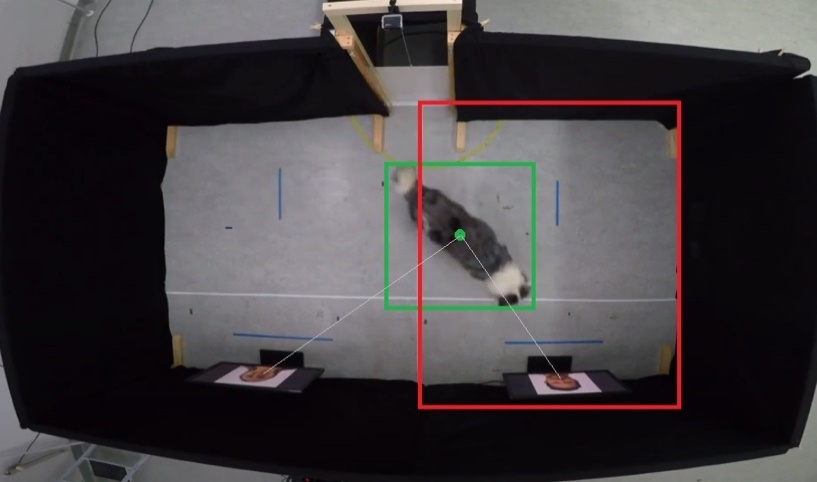


**Figure S6.** K9-Blyzer software detection of the dog’s centre of mass (marked by green dot) while the dog is located in the left AoI 1 (dog’s perspective; red frame). The dog’s distance to both screens is marked by the grey lines from the dog’s centre of mass to both screens.

To rule out an effect of the side on which the caregiver, stranger or familiar person was presented during the experiment trials, we checked whether there was a statistically significant difference for each of the measured parameters (AoI 1 left; AoI 1 right; AoI 2 left; AoI 2 right; dog’s distance to left screen; dog’s distance to right screen; FoV left; FoV right) in both experiments (counterbalanced left/ right side: Exp. 3a: stranger-caregiver, caregiver-stranger; Exp. 3b: familiar-caregiver, caregiver-familiar). While the majority of the data was approximately normally distributed (Shapiro-Wilk-test, *p*<0.01), given that the data subsets were uneven and several variables indicated large skewness, we adopted a non-parametric test to assess their independence (Mann-Whitney-U-test, *p*<0.05). All data analyses and data visualizations were conducted using *jamovi* (Version 1.2)^22^.

**RESULTS**

**Experiment 1 (fMRI task)**

**Emotion x attachment interaction described in more detail**

**Happy morph videos**

Regarding the happy morph videos, visual presentation of the happy caregiver compared to the happy familiar person was associated with increased activation in bilateral hippocampal regions, left caudal cingulate gyrus, left amygdala, left medial temporal lobe, right occipital lobe, and the rostral parietal lobe (see Table S3, section “happy: caregiver > familiar and Fig. 1b). Comparing visual presentation of the happy caregiver with the happy stranger resulted in increased activation in the right caudate nucleus, left insula, left rostral cingulate gyrus, occipital lobe, rostral and caudal left sylvian, and prorean gyrus regions (see Table S3, section “happy: caregiver > stranger Fig. 1b). Vice versa, visual presentation of the happy stranger (compared to the angry stranger) elicited increased activation only in a small number of clusters including the right caudate nucleus, left caudal composite gyrus and left thalamus (see Table S3, section “happy: caregiver < stranger). Comparing the happy stranger and happy familiar human models, the stranger revealed increased activation in brain regions such as the bilateral caudal and left medial regions of the sylvian gyrus, left rostral cingulate gyrus, caudal parietal regions, right olfactory tuberculum, right amygdala, and the piriform lobe (see Table S3, section “happy: familiar < stranger Fig. 1b). The reverse contrast resulted in increased activation within the right piriform lobe, left rostral suprasylvian gyrus, left caudate nucleus, and the right hippocampus (see Table S3, section “happy: familiar > stranger”). Visual presentation of the happy familiar (compared to the happy caregiver) elicited increased activation in brainstem regions, the right postcruciate gyrus, right thalamus, bilateral rostral sylvian gyrus regions, and the left cerebellum (see Table S3, section “happy: caregiver < familiar”).

**Angry morph videos**

Focusing on the visual presentation of human models with angry emotional facial expression, the angry caregiver (compared to the familiar) resulted in increased activation in the bilateral dorsal and right medial cingulate gyri, the right caudate nucleus, bilateral caudal sylvian gyrus regions, right dorsal temporal regions, the right cerebellum and the brainstem (see Table S3, section “angry: caregiver > familiar” Fig. 1c). Furthermore, comparing the angry caregiver against angry stranger, the caregiver elicited activation in the left caudate nucleus and thalamus (see Table S3, section “angry: caregiver > stranger”). The opposite contrast revealed increased activation in caudal frontal regions, the brainstem, the left cerebellum, the right splenial gyrus and left postcruciate gyrus (see Table S3, section “angry: caregiver < stranger” Fig. 1c).Visual presentation of the angry stranger (in comparison to angry familiar) elicited increased activation the left postcruciate gyrus, left hippocampus expanding into the left thalamus, left dorsal cingulate gyrus, right cerebellum and frontal and parietal regions (see Table S3, section “angry: familiar < stranger” Fig. 1c), whereby the opposing contrast revealed increased activation only in the right ectomarginal gyrus and brainstem (see Table S3, section “angry: familiar > stranger”).

|  | | | | | | | |
| --- | --- | --- | --- | --- | --- | --- | --- |
| Contrast, brain region & HRF | | | Coordinates | | | *z*-value | cluster size |
|  |  |  | x | y | z |  |  |
| **Happy > angry** | | |  |  |  |  |  |
| L hippocampus (T) | | -6 | -11 | 10 | 3.29 | 8 |  |
| **Happy < angry** | | |  |  |  |  |  |
| R parahippocampal gyrus (T) | | 11 | -14 | 1 | 3.34 | 11 |  |
| **Caregiver < familiar** | | |  |  |  |  |  |
| Encephalon (white matter) | | 11 | -5 | 6 | 3.93 | 6 |  |
| L cerebellum | | -10 | -30 | -8 | 3.82 | 11 |  |
| L caudal sylvian gyrus (T) | | -22 | -12 | 3 | 3.49 | 6 |  |
| R olfactory peduncle (F) | | 7 | 14 | 1 | 3.39 | 5 |  |
| R occipital gyrus (O) | | 14 | -35 | 3 | 3.38 | 6 |  |
| **Caregiver < stranger** | | |  |  |  |  |  |
| L rostral composite gyrus (F) | | -15 | 8 | 10 | 4.90 | 15 |  |
| L postcruciate gyrus (P) | | -12 | 3 | 13 | 4.29 | 14 |  |
| L pons | | -6 | -21 | -11 | 4.20 | 9 |  |
| Mesencephalon | | 2 | -12 | 8 | 3.61 | 6 |  |
| R rostral composite gyrus (F) | | 19 | 3 | 9 | 3.38 | 8 |  |
| Diencephalon | | 11 | -6 | 4 | 3.31 | 5 |  |
| Encephalon (white matter) | | 8 | 3 | 4 | 3.19 | 7 |  |
| L insula (T) | | -12 | -2 | 3 | 3.04 | 11 |  |
| R medial ectosylvian gyrus | | 13 | -14 | 13 | 3.03 | 7 |  |
| L caudal gyrus composite (T) | | -21 | -23 | -7 | 3.01 | 8 |  |
| L cerebellum | | -9 | -35 | -5 | 2.90 | 5 |  |
| L precruciate gyrus (F) | | -13 | 12 | 21 | 2.78 | 6 |  |
| **Familiar > stranger** | | | | | | | |
| Medulla oblongata | | 1 | -50 | -14 | 3.34 | 5 |  |
| L rostral composite gyrus (F) | | -12 | 11 | 13 | 3.05 | 5 |  |
| **Happy: caregiver < familiar** | | |  |  |  |  |  |
| R postcruciate gyrus (P) | | 10 | 0 | 18 | 4.13 | 18 |  |
| R thalamus (T) | | 5 | -8 | -1 | 3.69 | 10 |  |
| Medulla oblongata | | -1 | -44 | -16 | 3.56 | 10 |  |
| R rostral sylvian gyrus (T) | | 17 | -3 | 4 | 3.54 | 5 |  |
| Spinal medulla | | 1 | -54 | -13 | 3.54 | 5 |  |
| L cerebellum | | -12 | -30 | -10 | 3.49 | 7 |  |
| L caudal sylvian gyrus (T) | | -22 | -12 | 3 | 3.45 | 11 |  |
| L rostral composite gyrus (F) | | 16 | 5 | 10 | 3.37 | 5 |  |
| R rostral suprasylvian gyrus (T) | | 16 | 0 | 16 | 3.35 | 10 |  |
| R piriform lobe (T) | | 13 | -12 | -11 | 3.30 | 5 |  |
| **Happy: caregiver < stranger** | | |  |  |  |  |  |
| R caudate nucleus / white matter (T) | 8 | -2 | 6 | 3.62 | 9 |  |  |
| L caudal composite gyrus (T) | -21 | -21 | -7 | 3.24 | 5 |  |  |
| L thalamus | -3 | -8 | 4 | 3.06 | 5 |  |  |
| **Happy: familiar > stranger** | |  |  |  |  |  |  |
| R piriform lobe | 13 | -12 | -10 | 4.06 | 7 |  |  |
| L rostral suprasylvian gyrus (P) | -10 | -5 | 16 | 4.03 | 9 |  |  |
| L caudate nucleus / white matter (T) | -12 | -5 | 9 | 3.55 | 9 |  |  |
| R hippocampus (T) | 13 | -17 | -2 | 3.19 | 8 |  |  |
| **Angry: caregiver < familiar** | |  |  |  |  |  |  |
| R parahippocampal gyrus (T) | 10 | -20 | -8 | 3.83 | 14 |  |  |
| Pons | 1 | -23 | -16 | 3.80 | 7 |  |  |
| L rostral composite gyrus (F) | -13 | 11 | 12 | 3.53 | 5 |  |  |
| R caudal composite gyrus (T) | 16 | -20 | -8 | 3.22 | 11 |  |  |
| L insula (T) | -13 | -5 | 3 | 2.93 | 5 |  |  |
| L marginal gyrus (P) | -3 | -23 | 24 | 2.91 | 5 |  |  |
| **Angry: caregiver < stranger** | |  |  |  |  |  |  |
| L rostral composite gyrus (F) | -15 | 8 | 10 | 4.53 | 17 |  |  |
| L postcruciate gyrus (P) | -12 | 3 | 13 | 3.79 | 9 |  |  |
| R splenial gyrus (O) | 5 | -29 | 9 | 3.64 | 14 |  |  |
| Pons | 1 | -23 | -16 | 3.58 | 6 |  |  |
| L proreus gyrus (F) | -13 | 2 | 4 | 3.45 | 26 |  |  |
| Mesencephalon | 1 | -12 | -8 | 3.42 | 6 |  |  |
| Pons | -6 | -21 | -11 | 3.35 | 5 |  |  |
| Mesencephalon | -9 | -12 | -7 | 3.32 | 11 |  |  |
| L cerebellum | -9 | -30 | -10 | 3.03 | 5 |  |  |
| L gyrus rectus (F) | -4 | 18 | 3 | 3.00 | 6 |  |  |
| **Angry: familiar > stranger** | |  |  |  |  |  |  |
| R ectomarginal gyrus (T) | 10 | -26 | 18 | 3.59 | 7 |  |  |
| Pons | 4 | -24 | -7 | 3.35 | 8 |  |  |
| **Table S3.** Experiment 1 (fMRI): Task-related activation during visual stimulation.  Effects were tested for significance with a cluster-defining threshold of *p*< 0.005 uncorrected and a minimum cluster size of 5 voxels. The first local maximum within each cluster is reported; coordinates represent the location of peak voxels and refer to the canine breed-averaged template^4^. The template along with another single dog template^6^ served to determine anatomical nomenclature for all tables. These contrasts reported were not of interest for the present study but reported in light of full transparency, see Table 1 and 2 for contrasts most relevant for the present study; O, occipital lobe; T, temporal lobe; P, parietal lobe; O, occipital lobe; L, left; R, right. | | | | | | | |

**Experiment 2: Eye-tracking task**


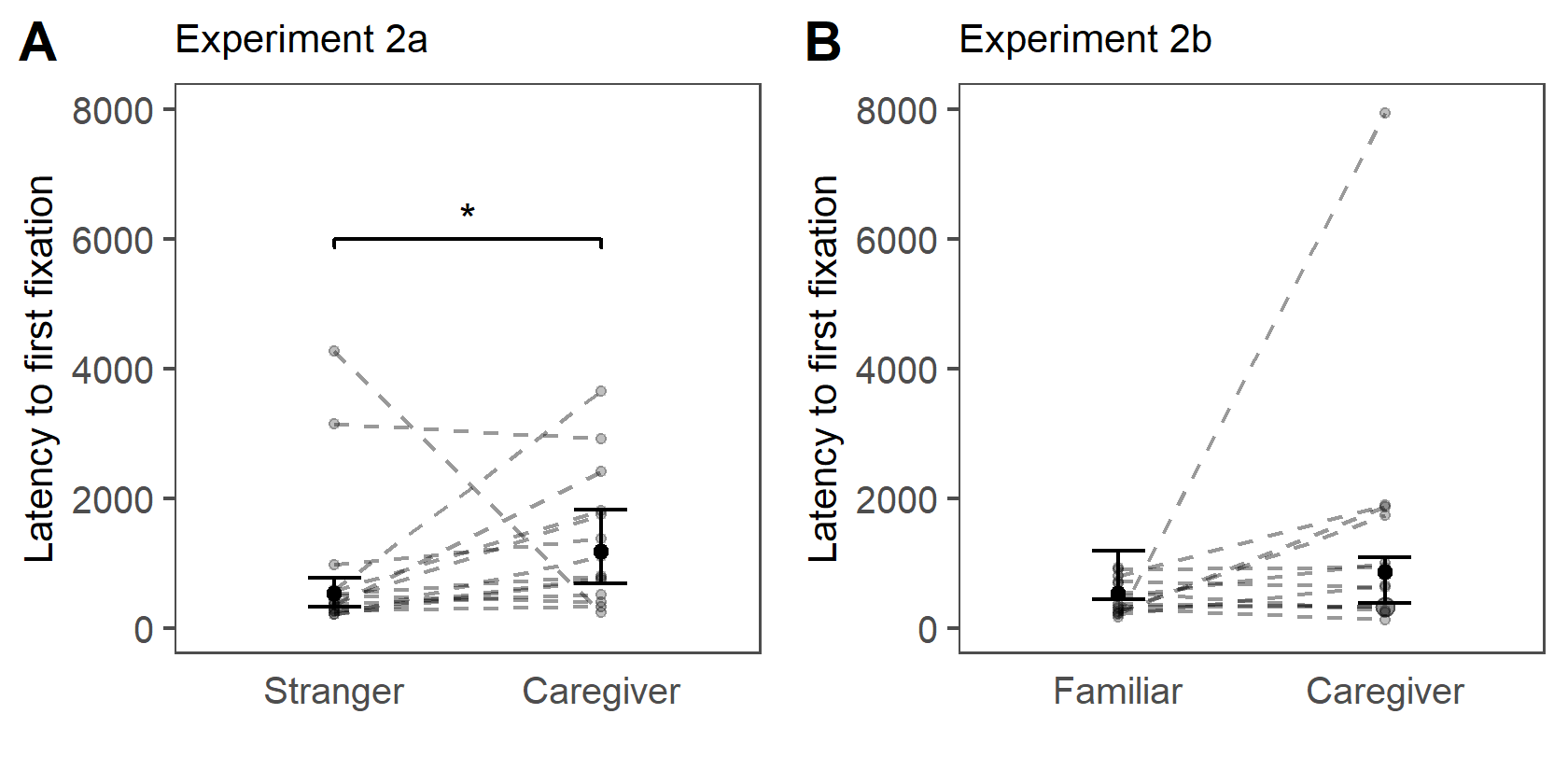


**Figure S7.** Relationship between the latency of the first fixation (in ms) into one of the two AoIs and the displayed stimuli in Experiment 2a (A) and 2b (B). GLMM: **p*<0.05. The grey points and dashed lines represent the individual looking patterns of each dog per experiment. The size of the points is proportional to the number of individuals. The bootstrapped 95% confidence intervals of the model are indicated by the vertical black lines.

| **term** | **estimate** | **SE** | **CI 95%** | | **χ²** | **df** | **P-value** | **Min^f^** | **Max^f^** |
| --- | --- | --- | --- | --- | --- | --- | --- | --- | --- |
| (Intercept) | 5.831 | 0.353 | 4.997 | 6.529 |  |  | NA^e^ | 5.703 | 5.958 |
| Emotion^b^ | 0.665 | 0.384 | -0.129 | 1.428 | 2.709 | 1 | 0.100 | 0.435 | 0.894 |
| Stimulus^c^ | 0.806 | 0.326 | 0.117 | 1.449 | 5.746 | 1 | 0.017 | 0.702 | 0.858 |
| Trial^e^ | 0.050 | 0.195 | -0.330 | 0.418 | 0.066 | 1 | 0.798 | -0.022 | 0.237 |
| Caregiver location^a^ | 0.206 | 0.320 | -0.461 | 0.867 | 0.412 | 1 | 0.521 | -0.001 | 0.352 |
| Age^d^ | 0.028 | 0.167 | -0.286 | 0.334 | 0.028 | 1 | 0.867 | -0.129 | 0.077 |

**Table S4.** Results of GLMM 2 investigating the latency of the first fixation in Exp. 2a

Note. Reference categories: ^a^caregiver location: left side, ^b^emotion: angry, ^c^stimulus: stranger; ^d^All covariates were z-transformed. ^e^not indicated because of limited interpretation. ^f^Minimum and maximum of the model estimates when dropping each case one at a time. The respective null model did not converge.


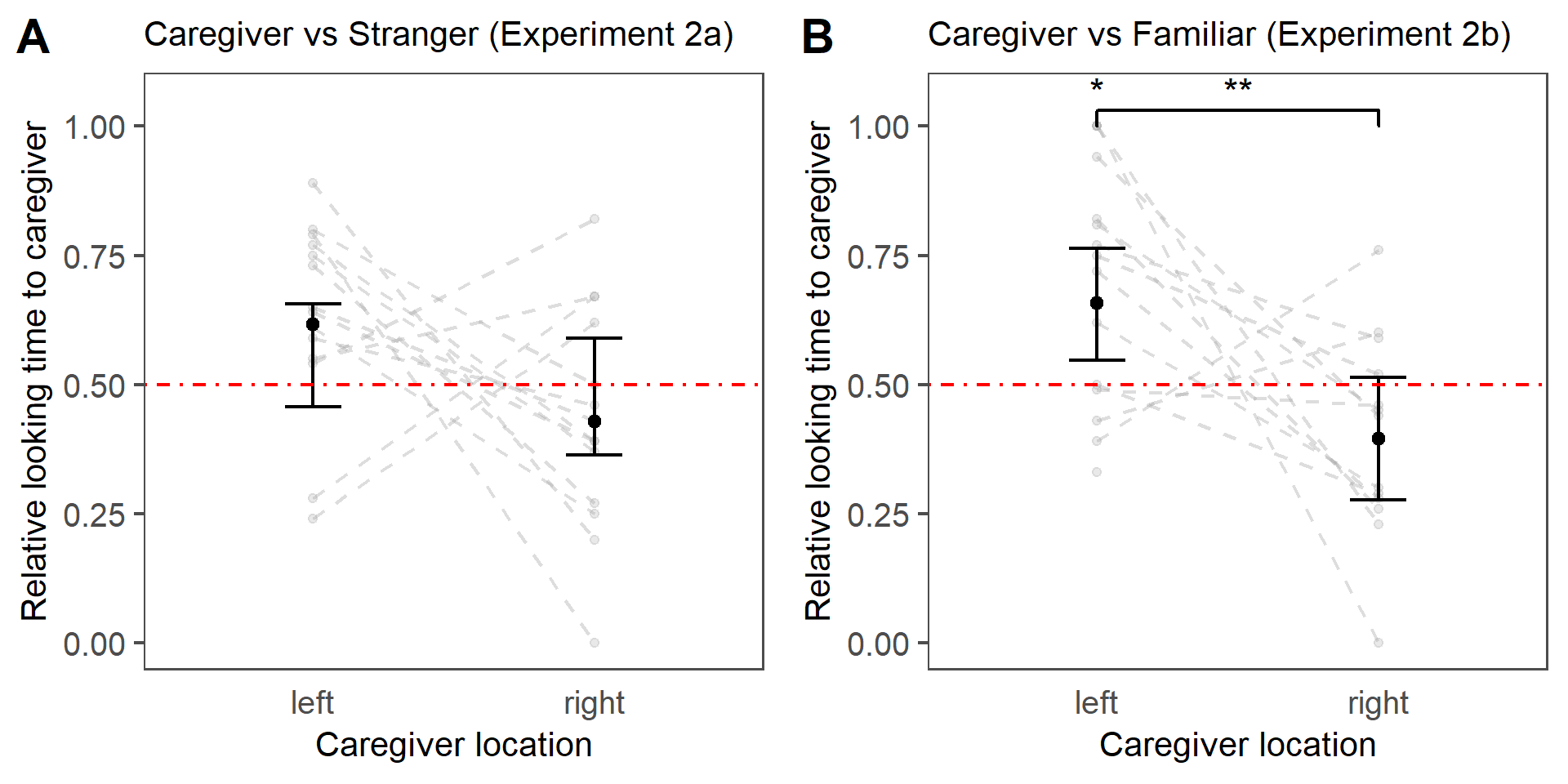


**Figure S8.** Relationship between the dogs’ relative looking time to the caregiver and the caregiver location on the screen (left or right side) in Experiment 2a (A) and 2b (B). The red dash-dotted line represents the chance level (0.5), GLMM: **p*<0.05, ***p*<0.01. The bootstrapped 95% confidence intervals of the model are indicated by the vertical black lines. The grey points and dashed lines represent the individual looking patterns of each dog per experiment.

| **term** | **estimate** | **SE** | **CI 95%** | | **χ²** | **df** | **P-value** | **Min^e^** | **Max^e^** |
| --- | --- | --- | --- | --- | --- | --- | --- | --- | --- |
| (Intercept) | 0.522 | 0.285 | -0.075 | 1.099 |  |  | NA^d^ | 0.425 | 0.763 |
| Caregiver location^a^ | -1.074 | 0.368 | -1.867 | -0.355 | 6.998 | 1 | 0.008 | -1.266 | -0.845 |
| Emotion^b^ | 0.259 | 0.322 | -0.445 | 0.954 | 0.644 | 1 | 0.422 | -0.054 | 0.364 |
| Trial^c^ | 0.030 | 0.169 | -0.312 | 0.355 | 0.031 | 1 | 0.860 | -0.110 | 0.090 |
| Age^c^ | -0.199 | 0.157 | -0.531 | 0.130 | 1.603 | 1 | 0.206 | -0.282 | -0.167 |

**Table S5.** Results of the GLMM 4 investigating the dwell time in Exp. 2b

Note. Reference categories: ^a^caregiver location: left side, ^b^emotion: angry; ^c^All covariates were z-transformed. ^d^not indicated because of limited interpretation. ^e^Minimum and maximum of the model estimates when dropping each case one at a time.


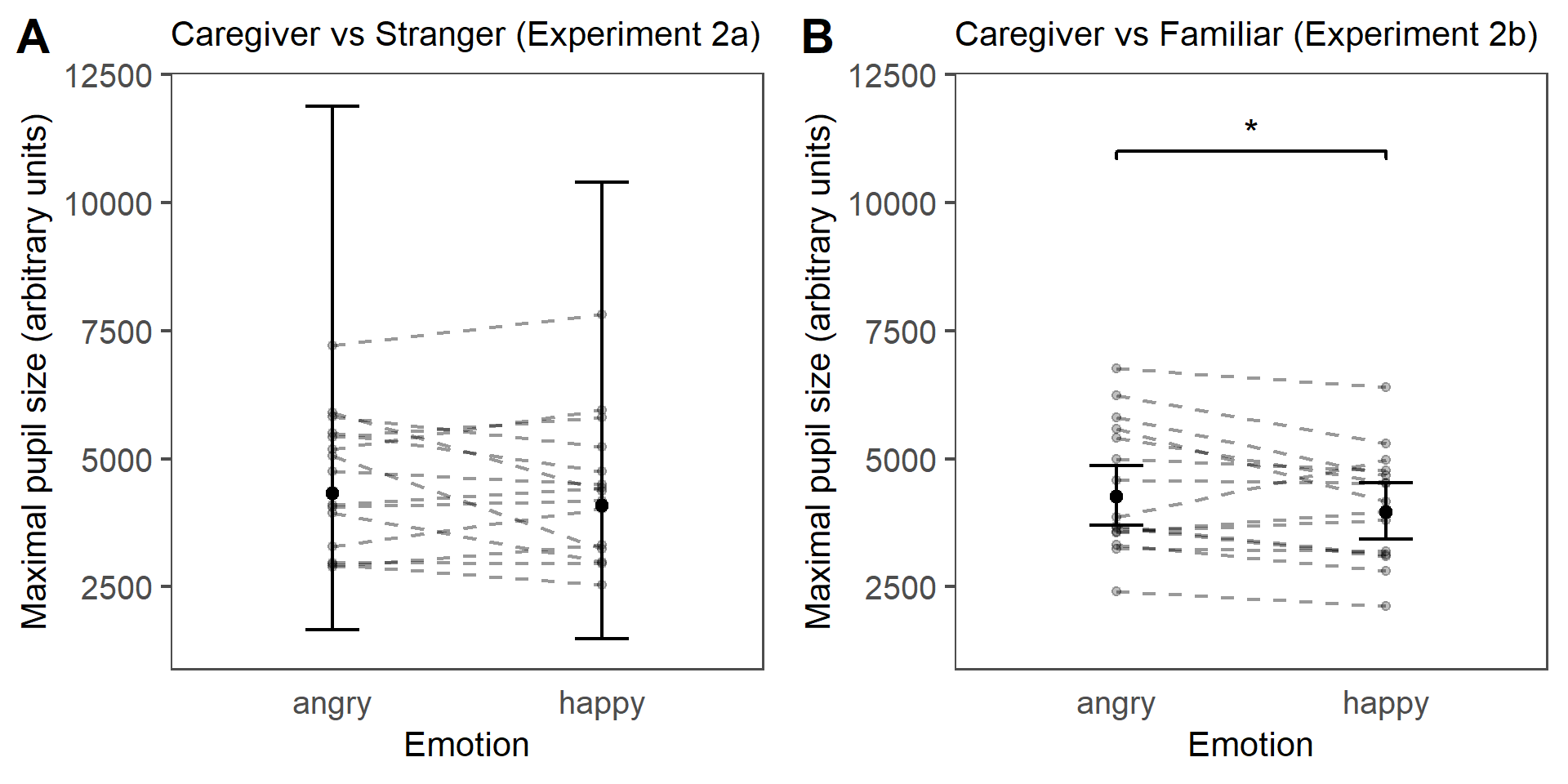


**Figure S9.** Relationship of the maximal pupil size of the dogs and the perceived emotional facial expressions in Experiment 2a (A) and 2b (B). GLMM: **p*<0.05. The bootstrapped 95% confidence intervals of the model are indicated by the vertical black lines. The grey points and dashed lines represent the individual looking patterns of each dog per experiment.

| **term** | **estimate** | **SE** | **CI 95%** | | **χ²** | **df** | **P-value** | **Min^f^** | **Max^f^** |
| --- | --- | --- | --- | --- | --- | --- | --- | --- | --- |
| (Intercept) | 8.353 | 0.073 | 8.223 | 8.492 |  |  | NA^d^ | 8.311 | 8.397 |
| Emotion^a^ | -0.074 | 0.035 | -0.147 | -0.003 | 4.225 | 1 | 0.040 | -0.095 | -0.054 |
| Stimulus^b^ | 0.006 | 0.029 | -0.048 | 0.063 | 0.041 | 1 | 0.839 | -0.011 | 0.023 |
| Trial^c^ | -0.019 | 0.018 | -0.058 | 0.018 | 1.055 | 1 | 0.304 | -0.032 | -0.013 |
| Age^c^ | -0.009 | 0.071 | -0.144 | 0.121 | NA^e^ |  | NA^e^ | -0.069 | 0.034 |

**Table S6.** Results of GLMM 6 investigating the maximal pupil size in Exp. 2b

Note. Reference categories: ^a^emotion: angry, ^b^stimulus: familiar person; ^c^All covariates were z-transformed. ^d^not indicated because of limited interpretation. ^e^Drop1 function of age did not converge. ^f^Minimum and maximum of the model estimates when dropping each case one at a time. The null model comprising only the control predictors age and trial number and the subject ID did not converge.

**Experiment 3: Behavioural preference task**

|  | | **Caregiver** | | | | **Stranger** | | | |
| --- | --- | --- | --- | --- | --- | --- | --- | --- | --- |
| **Area/ measure** | **Parameter** | ***median*** | ***mean*** | ***SD*** | ***counts*** | ***median*** | ***mean*** | ***SD*** | ***counts*** |
| AoI 1 | Time in area (in s) | 15.11 | 14.90 | 11.17 |  | 12.57 | 13.90 | 11.01 |  |
| AoI 2 | Time  in area (in s) | 0 | 2.30 | 4.56 |  | 0 | 1.92 | 4.19 |  |
| AoI 3 | Time in area (in s) | 0 | 0.58 | 2.67 |  | 0 | 0.49 | 1.69 |  |
| First choice | First AoI 2 entry (counts) |  |  |  | 25 |  |  |  | 27 |
| FoV | Time looking at screen (in s) | 0.83 | 2.11 | 3.39 |  | 0.71 | 2.03 | 3.60 |  |
| Dog-screen-distance | Average distance to screen (in pixels) | 302.10 | 303.40 | 78.33 |  | 303.50 | 317.6 | 70.67 |  |

**Table S7.** Descriptive statistics of time- and distance-related parameters of Exp. 3a

|  | | **Caregiver** | | | | **Familiar person** | | | |
| --- | --- | --- | --- | --- | --- | --- | --- | --- | --- |
| **Area/ measure** | **Parameter** | ***median*** | ***mean*** | ***SD*** | ***counts*** | ***median*** | ***mean*** | ***SD*** | ***counts*** |
| AoI 1 | Time in area (in s) | 16.98 | 15.08 | 11.68 |  | 11.87 | 13.92 | 11.88 |  |
| AoI 2 | Time in area (in s) | 0 | 1.39 | 4.51 |  | 0 | 1.84 | 4.74 |  |
| AoI 3 | Time in area (in s) | 0 | 0.46 | 2.95 |  | 0 | 0.42 | 2.99 |  |
| First choice | First AoI 2 entry (counts) |  |  |  | 18 |  |  |  | 14 |
| FoV | Time looking at screen (in s) | 0.46 | 1.37 | 1.94 |  | 0.12 | 1.54 | 2.91 |  |
| Dog-screen-distance | Average distance to screen (in pixels) | 299.94 | 305.40 | 69.59 |  | 307.33 | 303.6 | 69.32 |  |

**Table S8.** Descriptive statistics of time- and distance-related parameters of Exp. 3b

|  | **Exp. 3a: Caregiver-Stranger** | | | **Exp. 3b: Caregiver-Familiar person** | | |
| --- | --- | --- | --- | --- | --- | --- |
| **Area/ measure** | **U-value** | **Z-value** | **P-value** | **U-value** | **Z-value** | **P-value** |
| AoI 1 | 3246.5 | -0.63 | 0.52 | 3355 | 0.29 | 0.77 |
| AoI 2 | 3124.5 | -1.03 | 0.30 | 3162 | -0.91 | 0.36 |
| AoI 3 | 760.5 | 0.005 | 1 | 882 | 0.004 | 1 |
| FoV | 3313 | -0.42 | 0.67 | 3176 | 0.87 | 0.38 |
| Dog-screen-distance | 3206.5 | 0.77 | 0.44 | 3370 | -0.24 | 0.81 |

**Table S9.** Inferential statistics of time- and distance-related parameters of Experiment 3 (Mann-Whitney-U-Test)

**Supplementary Movie Captions**

**Movie S1.** Video showing a repetitively presented morph video of the portrait of a subjects’ caregiver transforming from a neutral to a happy facial expression. The expression transformation is shown three times in a row as in the eye-tracking task (Experiment 2; 5x3 s).

**Movie S2.** Video showing a repetitively presented morph video of the portrait of a subjects’ caregiver transforming from a neutral to an angry facial expression. The expression transformation is shown three times in a row as in the eye-tracking task (Experiment 2; 5x3 s).

**References**

1. Horn, L., Range, F. & Huber, L. Dogs’ attention towards humans depends on their relationship, not only on social familiarity. *Anim. Cogn.* **16**, 435–443 (2013).

2. Willenbockel, V. *et al.* Controlling low-level image properties: The SHINE toolbox. *Behav. Res. Methods* **42**, 671–684 (2010).

3. Sladky, R. *et al.* Slice-timing effects and their correction in functional MRI. *Neuroimage* **58**, 588–594 (2011).

4. Nitzsche, B. *et al.* A stereotaxic breed-averaged, symmetric T2w canine brain atlas including detailed morphological and volumetrical data sets. *Neuroimage* **187**, 93–103 (2019).

5. Yushkevich, P. A. *et al.* User-guided 3D active contour segmentation of anatomical structures: Significantly improved efficiency and reliability. *Neuroimage* **31**, 1116–1128 (2006).

6. Czeibert, K., Andics, A., Petneházy, Ö. & Kubinyi, E. A detailed canine brain label map for neuroimaging analysis. *Biol. Futur.* **70**, 112–120 (2019).

7. Boch, M. *et al.* Tailored haemodynamic response function increases detection power of fMRI in awake dogs (Canis familiaris). *Neuroimage* **224**, 117414 (2020).

8. Karl, S., Boch, M., Virányi, Z., Lamm, C. & Huber, L. Training pet dogs for eye-tracking and awake fMRI. *Behav. Res. Methods* 1–19 (2019). doi:10.3758/s13428-019-01281-7

9. Barber, A. L. A., Randi, D., Müller, C. A. & Huber, L. The processing of human emotional faces by pet and lab dogs: Evidence for lateralization and experience effects. *PLoS One* **11**, 1-22: e0152393 (2016).

10. Baayen, R. H. *Analyzing linguistic data: a practical introduction to statistics using R*. (Cambridge University Press, 2008).

11. Barr, D. J. Random effects structure for testing interactions in linear mixed-effects models. *Front. Psychol.* **4**, 3–4 (2013).

12. Schielzeth, H. & Forstmeier, W. Conclusions beyond support: Overconfident estimates in mixed models. *Behav. Ecol.* **20**, 416–420 (2009).

13. Bolker, B. M. *Ecological models and data in R*. (Princeton University Press, 2008).

14. Dobson, A. J. *An introduction to generalized linear models*. (Chap- man & Hall/CRC press, 2002).

15. Forstmeier, W. & Schielzeth, H. Cryptic multiple hypotheses testing in linear models: Overestimated effect sizes and the winner’s curse. *Behav. Ecol. Sociobiol.* **65**, 47–55 (2011).

16. R Core Team, R. R: A Language and Environment for Statistical Computing. (2019).

17. Brooks, M. E. *et al.* glmmTMB balances speed and flexibility among packages for zero-inflated generalized linear mixed modeling. *R J.* **9**, 378–400 (2017).

18. Bates, D., Mächler, M., Bolker, B. M. & Walker, S. C. Fitting linear mixed-effects models using lme4. *J. Stat. Softw.* **67**, (2015).

19. Amir, S., Zamansky, A. & van der Linden, D. K9-Blyzer – Towards Video-Based Automatic Analysis of Canine Behavior. in *Proceedings of the Fourth International Conference on Animal-Computer Interaction* 1–5 (2017). doi:10.1145/3152130.3152142

20. Bleuer-Elsner, S. *et al.* Computational Analysis of Movement Patterns of. *Animals* **9**, 1140 (2019).

21. Mathis, A. *et al.* DeepLabCut: markerless pose estimation of user-defined body parts with deep learning. *Nat. Neurosci.* **21**, 1281–1289 (2018).

22. The jamovi project (2020). jamovi (Version 1.2) [Computer Software]. Retrieved from https://www.jamovi.org. (2020).
